# EWS::FLI1-DHX9 interaction promotes Ewing sarcoma sensitivity to DNA topoisomerase 1 poisons by altering R-loop metabolism

**DOI:** 10.1038/s41388-025-03496-9

**Published:** 2025-07-28

**Authors:** Joaquin Olmedo-Pelayo, Esperanza Granado-Calle, Daniel Delgado-Bellido, Laura Lobo-Selma, Carmen Jordan-Perez, Ana T. Monteiro-Amaral, Anna C. Ehlers, Shunya Ohmura, Daniel J. Garcia-Dominguez, Carlos Mackintosh, Angel M. Carcaboso, Javier Alonso, Isidro Machado, Antonio Llombart-Bosch, Katia Scotlandi, Thomas G. P. Grünewald, Fernando Gomez-Herreros, Enrique de Alava

**Affiliations:** 1 Instituto de Biomedicina de Sevilla, IBiS/Hospital Universitario Virgen del Rocío/CSIC/Universidad de Sevilla, Seville, Spain; 2 Centro de Investigación Biomédica en Red de Cáncer (CIBERONC), Instituto de Salud Carlos III, Madrid, Spain; 3 Departamento de Citología e Histología Normal y Patológica, Universidad de Sevilla, Seville, Spain; 4 Departamento de Genética, Universidad de Sevilla, Seville, Spain; 5 Center for Neurosciences and Cell Biology, Center for Innovative Biomedicine and Biotechnology (CIBB), Faculty of Medicine (Polo 1), University of Coimbra, Coimbra, Portugal; 6 Division of Translational Pediatric Sarcoma Research, German Cancer Consortium (DKTK), German Cancer Research Center (DKFZ), Heidelberg, Germany; 7 Hopp Children’s Cancer Center (KiTZ), Heidelberg, Germany; 8 Departamento de Bioquímica Médica y Biología Molecular e Inmunología, Universidad de Sevilla, Seville, Spain; 9 Predictive Sciences, Bristol Myers Squibb’s Center for Innovation and Translational Research Europe (CITRE), Seville, Spain; 10 SJD Pediatric Cancer Center Barcelona, Hospital Sant Joan de Deu, Barcelona, Spain; 11 Pediatric Cancer Program, Institut de Recerca Sant Joan de Deu, Barcelona, Spain; 12 Unidad de Tumores Sólidos Infantiles, Instituto de Investigación de Enfermedades Raras, Instituto de Salud Carlos III, Madrid, Spain; 13 Centro de Investigación Biomédica en Red de Enfermedades Raras (CIBERER), Instituto de Salud Carlos III, Madrid, Spain; 14 Pathology Department, Instituto Valenciano de Oncología, Valencia, Spain; 15 Patologika Laboratory, Hospital QuirónSalud, Valencia, Spain; 16 Pathology Department, University of Valencia, Valencia, Spain; 17 Laboratory of Experimental Oncology, IRCCS Istituto Ortopedico Rizzoli, Bologna, Italy; 18 Institute of Pathology, Heidelberg University Hospital, Heidelberg, Germany

**Keywords:** Paediatric cancer, Sarcoma

## Abstract

Drug resistance is an ill-defined cause of dismal outcomes in cancer. Ewing sarcoma (EwS), a pediatric cancer characterized by high therapy failure rates, is driven by a single oncogenic event generating *EWSR1*::*ETS* gene fusions (primarily *EWSR1::FLI1*) in a silent genomic background. This provides a straightforward model to study the impact of gene fusions on drug responses. Here, we describe a novel mechanism of sensitivity to DNA topoisomerase 1 poisons in EwS. We discovered that EWS::FLI1 prevents the resolution of R-loops induced by these drugs via sequestering DHX9 helicase, ultimately resulting in R-loop accumulation, replication stress, and genome instability. In turn, excessive DHX9 or reduced EWS::FLI1 levels render EwS cells resistant to the active metabolite of irinotecan (SN-38) independent of proliferation and global transcription rates. This resistance helps explain how elevated DHX9 levels predict worse clinical outcomes. Overall, our research demonstrates the impact of a dominant mutation on cancer drug sensitivity, highlighting its significant clinical implications.

## Introduction

Ewing sarcoma (EwS) is an aggressive mesenchymal neoplasm mainly occurring in children, adolescents, and young adults. Although 5-year survival for patients with localized disease is ~70–80%, the prognosis remains poor for those with metastatic disease, with long-term survival rates around 30% [1]. In fact, 25–30% of patients present metastasis at diagnosis [2, 3]. Despite the collective efforts to implement novel therapeutic approaches, current EwS treatment remains based on combining traditional chemotherapy, radiotherapy, and surgery [4]. The prevailing first-line chemotherapy for EwS is the VDC/IE regimen, which includes vincristine, doxorubicin, and cyclophosphamide alternating with ifosfamide and etoposide [5, 6]. Despite EwS being highly sensitive to genotoxic agents, therapy failure and tumor relapse occur in 30–35% of patients with primary localized disease and 50–80% of the cases with metastasis [4]. Recently, other genotoxic agents (e.g., irinotecan and temozolomide) have been successfully evaluated in clinical trials as effective treatment options in relapsed or advanced refractory EwS [7, 8].

Genetically, EwS is characterized by pathognomonic gene fusions between TET and ETS gene family members, mainly affecting *EWSR1* and *FLI1* genes (in 85% of the cases) [1, 9]. Although EWS::FLI1 is the established main driver of EwS, its contribution to the hypersensitivity of this tumor to genotoxic agents has not been extensively addressed. Strikingly, *Gorthi and colleagues* recently described a novel mechanism of drug sensitivity involving the EWS::FLI1-mediated accumulation of R-loops [10]. They proposed that R-loops-dependent recruitment of BRCA1 to the elongating transcription machinery would reduce the homologous recombination repair (HR) capacity of EwS cells, giving rise to the ‘BRCAness’ phenotype previously associated with EwS tumors.

R-loops are three-stranded nucleic acid structures generated during transcription and composed of a DNA:RNA hybrid and a displaced single-stranded DNA [11]. Despite its physiological roles in cellular processes such as DNA replication or gene expression, R-loops accumulation is a source of genome instability [12, 13]. Different mechanisms finely regulate R-loop levels, including RNA processing factors, topoisomerases, and chromatin remodelers [14]. Additionally, enzymes that degrade the RNA component of R-loops, such as RNAse H1 and RNAse H2 [15], and enzymes with DNA/RNA helicase activity, such as DEAD-Box RNA helicases or DEAH helicase DHX9 have been implicated in R-loops metabolism [16, 17].

DHX9 (also known as RNAse helicase A (RHA)) is an NTP-dependent helicase with DNA/RNA helicase activity involved in different cellular processes such as DNA replication, transcription, or RNA processing [18]. The role of DHX9 in R-loop metabolism is not completely understood. The impact of DHX9 on R-loop accumulation varies depending on the cellular environment; it can either increase or decrease R-loop levels. On the one hand, the DHX9-mediated unwinding of nascent RNA promotes R-loop formation when splicing is impaired, and the interaction of DHX9 and elongating RNAPII is prolonged along gene bodies [19]. On the other hand, DHX9 has also been implicated in the resolution of both physiological and DNA topoisomerase 1 (TOP1) poisoning-induced R-loops, preventing the generation of genome instability [17, 20, 21]. Notably, DHX9 interacts with EWS::FLI1 as a transcriptional cofactor, enhancing EWS::FLI1-associated aberrant transcription [22, 23].

In this work, we have studied the mechanism underlying R-loop accumulation in EwS cells and its potential implication in the sensitivity to genotoxic agents. We demonstrate that EwS known hypersensitivity to TOP1 poison SN-38, the active molecule of irinotecan, depends on the interaction between EWS::FLI1 and DHX9. Our findings indicate that recovering DHX9 activity enhances R-loop resolution in EwS, reducing drug-associated replication stress, DNA damage and cytotoxicity. These results offer a possible explanation for the link between R-loop accumulation and increased drug sensitivity in EwS.

## Methods

### Cell lines and culture conditions

Human EwS: A4573, A-673, TC-71, and EW-7; human breast cancer: MBA-MB-436 and HCC-1937, human fibroblast MRC-5, and sarcoma-derived U2OS, HT-1080, SK-UT-1, and 93T449 cell lines were purchased from ATCC. A673/TR/shEF cell line was gently provided by Dr. J. Alonso (Madrid, Spain). TC-71/TR/shEF cell line was gently provided by Dr. T. Grünewald (Heidelberg, Germany). The HeLa EF model was previously generated by our group [24]. All cell lines were grown at 37 °C, 5% CO_2_, routinely tested for mycoplasma contamination using the MycoAlert Detection Kit (Lonza Group Ltd, LT07-318), and authenticated by STR analysis (CLS, Cell Lines Service GmbH). Cell line characteristics, origin, and culture conditions are depicted in Supplementary Table 2. A673/TR/shEF, TC-71/TR/shEF and HeLa EF cells were incubated with 1 µg/ml doxycycline (DOX)(Sigma, D9891) for the indicated times. Drugs used in this study are listed in Supplementary Table 3.

### Patient-derived xenograft (PDX) models

EwS (IEC-73) and non-EwS (osteosarcoma, IEC-036; undifferentiated pleomorphic sarcoma, IEC-056) PDXs were previously stablished by our group. HSJD-ES-002 and HSJD-ES-006 models were gently provided by Dr. A. Carcaboso (Barcelona, Spain). Tumors were subcutaneously implanted in 6-week-old athymic-nude (nu/nu) female mice (Envigo) in both flanks. When tumor volume reached a size of around 150 mm^3^, mice were randomized into two groups, and 10 mg/kg irinotecan (Accord Healthcare, 713386.5) was injected intraperitoneally five times per week for 2 weeks [(dx5)x2]. Animals were randomly selected based on predefined inclusion criteria to ensure unbiased group allocation. The investigator was not blinded to group allocation throughout the experiment. The control group was treated with a physiological saline solution. Tumor size and mouse weight were measured every 2 days. Tumor volume was calculated as *V* = (*a* × *b*^2^ × p)/6, where *b* is the smallest measure and *a* the largest measure. Part of the animals were sacrificed on day 7 of treatment and tumors were embedded in paraffin for the evaluation of tumoral necrosis. Hematoxylin/Eosin staining was performed according to conventional protocols and evaluated by an experienced pathologist. When tumor volume reached the predefined humane endpoint of around 1200 mm^3^ (=event), animals were sacrificed by cervical dislocation. Animal experiments were approved by the Consejeria de Agricultura, Pesca, Agua y Desarrollo Rural; Junta de Andalucia (08-05-2018-082) and performed according to protocols and conditions reflected in the European guidelines (EU Directive 2010/63/EU). PDXs characteristics are shown in Supplementary Table 1.

### Plasmid and siRNA transfection

Transient cell transfections with plasmids and siRNAs (listed in Supplementary Table 4) were performed using jetPRIME (Polyplus, 101000046) according to the manufacturer protocol.

### Analysis of cell cycle and apoptosis

For the analysis of cell cycle, cells were washed twice with PBS and fixed with 70% ethanol overnight at 4 °C. Then, cells were washed twice with PBS and incubated with propidium iodide (PI) solution (100 µg/ml PI and 100 µg/ml RNAse A) for 30 min. Finally, cells were washed with PBS before FACS acquisition. For the analysis of apoptosis, cells and supernatants were collected and washed twice with PBS. After that, cells were incubated with AnnexinV and PI (Immunostep; FITC-conjugated, ANXVKF7-100T or DY634-conjugated, ANXVKDY-100T) according to the manufacturer’s instructions for 15 min. 10,000–20,000 cells were counted for each sample using a BD FACSCanto II flow cytometer (Becton Dickinson). Data were analyzed using FlowJo v.10.2 software, and total AnnexinV-positive cells (early + late apoptosis) were shown.

### Metaphase spreads and sister chromatid exchanges (SCEs) assay

SCEs assay was performed as previously described by our group [25]. Cells were incubated with 10 μM BrdU (Sigma-Aldrich, B5002) for 40 h. Then, cells were treated with 2.5 µM etoposide for 30 min and washed twice with PBS. After treatment, cells were cultured in drug-BrdU-free, 0.2 μg/ml demecolcide-containing media (Sigma-Aldrich, D1925) for 8–14 h. Cells were collected and incubated in 0.03 M trisodium citrate solution for 30 min at 37 °C. After that, cells were fixed in 3:1 methanol:acetic acid. Fixed cells were dropped onto acetic acid-humidified slides and incubated in 10 μg/ml bisbenzimide solution (Hoechst 33258, Sigma-Aldrich, 14530) for 20 min. Next, slides were washed in 20× SSC buffer and irradiated in a 365 nm UV-lamp for 45 min. Finally, slides were incubated in 20× SSC at 65 °C for 1 h and stained for 20 min with 5% Giemsa solution (Sigma-Aldrich, GS500).

### DRGFP reporter plasmid assay

Cells were grown on 6-well plates and transfected with 1 µg DRGFP, 1 µg CBASceI, and 0.2 µg pmCherry-N1 (used as a control of transfection) plasmids. Where indicated, cells were incubated with 10 µM DNA-PK inhibitor NU-7441 for 24 h. After 48 h of transfection, cells were washed twice with PBS, detached, and inspected using a BD FACSCanto II flow cytometer. Analysis of FACS data was performed using FlowJo v.10.2 software. Data show the percentage of GFP-positive with respect to mCherry-positive cells.

### RNA extraction, reverse transcription, and quantitative real-time PCR

Total RNA was extracted from cultured cells using the miRNeasy Kit (Qiagen, 217084) according to the manufacturer’s instructions. DNA was removed using RQ1 DNase (Promega, M6101). RNA quantity was measured using a Nanodrop ND-2000 Spectrophotometer (Thermo Scientific). 500–1000 ng of RNA were reverse transcribed using MultiScribe Reverse Transcriptase Kit (Invitrogen, 4311235) according to the manufacturer’s protocol. qPCR was performed on a 7900HT Fast Real-Time PCR system (Applied Biosystems) using TaqMan probes and TaqMan Universal PCR Master Mix (Applied Biosystems, 4326708), or primers and iTaq Universal SYBR Green Supermix (Biorad, 1725270). TaqMan probes and primers are listed in Supplementary Tables 5 and 6, respectively. qPCR data were analyzed using ExpressionSuite software v1.3 (Thermo Fisher Scientific). 2^−ΔCt^ method was used to calculate relative gene expression levels, where ΔCt corresponds to the difference between the Ct of the target gene and the Ct of GAPDH.

### EdU and EU incorporation assay

Cells were grown on coverslips and incubated with 0.5 mM EU for 45 min (BaseClick, BCN-003) or 10 µM EdU for 20 min (BaseClick, BCN-001). Next, coverslips were washed twice with PBS and fixed with 4% PFA (Thermo Scientific, 28908) for 10 min at room temperature. Cells were permeabilized with PBS-0.2% Triton X-100 for 10 min and blocked with PBS-5% BSA for 1 h. After washout, cells were incubated with the Reaction cocktail solution (100 mM Tris-HCl pH 8.5, 1 mM CuSO_4_, 1 µM Fluorescein Azide 6-FAM (BaseClick, BCFA-001), 100 mM ascorbic acid) for 30 min at room temperature, under agitation and in the dark. After incubation, coverslips were washed twice with PBS-1% BSA and 4 times with PBS-0.1% Tween 20 for 10 min. Finally, cells were counterstained with DAPI (Sigma-Aldrich, 09542) for 5 min and mounted using Dako Fluorescent Mounting Medium. Images were acquired using Olympus BX61 Fluorescence Motorized Microscope (Olympus) and analyzed using ImageJ software version 1.54.

### Immunocytochemistry (ICC) and Immunohistochemistry (IHC)

For ICC, cell lines were collected, and pellets were fixed in 25% formalin before inclusion in paraffin. ICC and IHC were performed at HUVR-IBiS Biobank facility according to conventional protocols using antibodies listed in Supplementary Table 7. Briefly, 4 µm tissue sections from paraffin blocks were dewaxed in xylene and rehydrated in a series of graded alcohols. Antigen retrieval was performed with a PT link instrument (Agilent) using EDTA buffer (pH 8.0). After that, sections were incubated in H_2_O_2_ for 30 min and blocked with 1% blocking solution (Roche) in PBS-0.05% Tween-20. After primary antibodies, sections were incubated with peroxidase-labeled secondary antibodies and 3,3-diaminobenzimide, according to manufacturer's protocol (Leica Biosystems). Slides were counterstained with Hematoxylin and mounted using DPX (BDH Laboratories). Samples were evaluated by an experienced pathologist and scored according to the staining intensity (I) in: negative (I = 0), low (I ≤ 1), medium (I > 1 and ≤2) and high (I > 2).

### Western blotting

Western blotting was performed as previously described by our group [26]. Briefly, cell lines were lysed using RIPA buffer (10 mM Tris-HCl pH 8, 1 mM EDTA, 0.5 mM EGTA, 1% Triton X-100, 0.1% Sodium Deoxycholate, 0.1% SDS, 140 mM NaCl), supplemented with protease (Roche, 11697498001) and phosphatase inhibitors (1 mM Na_3_O_4_V (Sigma-Aldrich, 450243)) and 1 mM NaF (Sigma-Aldrich, S1504)). Protein concentration was determined using Pierce^TM^ BCA Protein Assay Kit (Thermofisher, 23225). Primary antibodies (listed in Supplementary Table 7) were blocked in 5% BSA TBS-0.1% Tween-20. HPRT-conjugated anti-mouse (Biorad, 1706516) and anti-rabbit (Biorad, 1706515) secondary antibodies were incubated for 1 h at room temperature (1:5000). Images were captured using Chemidoc Imaging System (Bio-Rad) and band density was determined using Image Lab software 6.1 (Bio-Rad).

### Slot blot

Slot blot was carried out as previously described [27], with modifications. Cells were lysed overnight at 37 °C by using SDS/Proteinase K standard method. Next, nucleic acids were extracted by phenol/chloroform and EtOH/sodium acetate purification. Pellets were resuspended in Milli-Q water and sonicated in a Bioruptor (Diagenode) for three cycles at high intensity. 1 µg of DNA was treated with 0.1 ng of RNAse A (Invitrogen, AM2271) at 37 °C for 2 h. For RNAse H1 controls, 1 µg of DNA was incubated with 6U RNAse H (NEB, M0297) at 37 °C for 2 h. Then, samples were loaded into pre-humidified Hybond-N+ Nylon membranes (Amersham, RPN303B) using a slot blot apparatus (Hoefer, PR648). Membranes were UV-crosslinked (0.12 J/m^2^), blocked with TBST-5% non-fat milk at room temperature for 1 h, and incubated with 1:1000 s9.6 antibody (Kerafast, ENH001) overnight at 4 °C. After washes, membranes were incubated with 1:5000 HPRT-conjugated anti-mouse secondary antibody (Biorad, 1706516) for 1 h at room temperature. After image acquisition using Chemidoc Imaging System (Bio-Rad), membranes were incubated with 0.02% Methylene blue in 0.3 M sodium acetate (pH 5.2) for 10 min to stain total DNA. Band density was determined using the Image Lab software 6.1 (Bio-Rad). Data shows s9.6 signal normalized to Methylene blue staining.

### Coimmunoprecipitation (co-IP)

For RNAPII-Ser2p immunoprecipitation, cells were grown in 100 mm cell culture dishes up to 80–90% of confluency. Then, cells were washed twice with ice-cold PBS and collected through scrapping. 10% of cells (WCE) were lysed in 2× LB (125 mM Tris-HCl pH 6.8, 4% SDS, 0.02% bromophenol blue, 20% glycerol, 200 mM DTT) and maintained on ice. The rest of the cells were lysed with Lysis Buffer 1 (250 nM sucrose, 50 mM Tris-HCl, 5 mM MgCl_2_, 0.25% NP40) on ice for 10 min. After centrifugation, nuclei were washed with Buffer 2 (1 M sucrose, 50 mM Tris-HCl, 5 mM MgCl_2_) and lysed using Nuclear Buffer Lysis 3 (20 mM Tris-HCl, 0.4 M NaCl, 15% glycerol, 1.5% Triton X-100, 100 mM CaCl_2_) supplemented with protease (Roche, 11697498001) and phosphatase inhibitors (1 mM Na_3_O_4_V (Sigma-Aldrich, 450243)) and 1 mM NaF (Sigma-Aldrich, S1504)). Lysates were sonicated in a Bioruptor (Diagenode) for 2 cycles at high intensity, and incubated overnight under rotation at 4 °C with 5 µg/ml of anti-RNAPII-Ser2p (Abcam, ab193468) or anti-IgG (Cell Signaling, 3900). Next, extracts were incubated with Dynabeads^TM^ Protein G (Invitrogen^TM^, 10004D) for 2 h under rotation at 4 °C. After washes, beads were resuspended in 2× LB and boiled at 99 °C for 5 min. For DHX9:GFP immunoprecipitation, cells were collected upon 48 h DHX9:GFP or control plasmid transfection. Then, cells were lysed in Lysis Buffer (25 mM HEPES pH 8, 150 mM NaCl, 10% Glycerol, 0.5% Triton X-100) and sonicated for 3 cycles at low intensity. Protein extracts were incubated with ChromoTek GFP-Trap Agarose beads (ProteinTech, GTA-20) under rotation for 2 h at 4 °C. After washes, beads were resuspended in 2× LB and boiled at 99 °C for 5 min. Western blotting was performed as previously described.

### Immunofluorescence

Cells were cultured on coverslips for 24 h before treatment or transfection. After that, slides were washed twice with PBS and fixed with 4% PFA for 10 min at room temperature. Next, cells were permeabilized with PBS-0.2% Triton X-100 for 10 min, blocked with PBS-5% BSA for 1 h, and incubated with primary antibodies for 1–3 h in PBS-1% BSA. Primary antibodies used in this study are listed in Supplementary Table 7. Slides were washed twice with PBS-1% BSA for 10 min and incubated with corresponding Cy2/Cy3-conjugated secondary antibodies (Jackson Laboratories) for 1 h. After washes, cells were counterstained with DAPI for 5 min and mounted using Dako Fluorescent Mounting Medium (DAKO, S3023). Images were taken using Olympus BX61 Fluorescence Motorized Microscope (Olympus). Fluorescence nuclear intensity was obtained using ImageJ software version 1.54. DAPI signal was used to delimit the nuclei.

### Proximity ligation assay (PLA)

PLA was performed using Duolink In Situ Detection Reagents Red kit (Sigma-Aldrich, DUO92008), according to the manufacturer’s instructions. Briefly, cells grown on coverslips were fixed with 4% PFA for 10 min at room temperature and permeabilized with PBS-0.2% Triton X-100 for 10 min. Primary antibodies (listed in Supplementary Table 7) were incubated for 1.5 h at room temperature. Finally, cells were counterstained with DAPI for 5 min and mounted using Dako Fluorescent Mounting Medium. For EWS::FLI1-HA/RNAPII-Ser2p PLA, coverslips were additionally incubated with Cy2-conjugated anti-mouse secondary antibody (1 h), for the detection of *EWSR1::FLI1* overexpressing cells.

### DNA fiber assay

DNA fiber assay was performed as previously described [28]. Briefly, cells were labeled with 25 µM 5-Chloro-2′-deoxyuridine (CldU, Sigma-Aldrich, CAS 50-90-8) for 30 min. After washes, cells were incubated with 50 µM 5-Iodo-2′-deoxyuridine (IdU, Sigma-Aldrich, CAS 54-42-2) and 5 µM SN-38 for 30 min. Then, cells were collected and dropped on frosted slides. Immediately, cells were lysed with Spreading Buffer (200 mM Tris-HCl pH 7.5, 50 mM EDTA pH 8, 0.5% SDS) and fixed with 3:1 methanol:acetic acid solution. Next, DNA was denatured with 2.5 M HCl for 1 h and incubated with 1:1000 rat anti-BrdU (Abcam, ab6326) for 1 h at 4 °C, 1:500 donkey anti-rat Alexa Fluor 488 secondary antibody (Thermofisher, A21208) for 1.5 h at 4 °C, 1:500 mouse anti-BrdU (BD biosciences, 347580) overnight at 4 °C and, finally, 1:250 goat anti-mouse Alexa Fluor 546 secondary antibody (Thermofisher, A11003) for 2 h at 4 °C. Montage was performed using Fluoromount-G Mounting Medium (eBioscience, 00-4958-02). Images were acquired using Olympus BX61 Fluorescence Motorized Microscope (Olympus) and analyzed using ImageJ software version 1.54.

### Chromatin immunoprecipitation (ChIP-qPCR)

ChIP experiments were performed using the SimpleChIP Enzymatic Chromatin IP Kit (Cell Signaling, 9003) according to manufacturer’s instructions. Cells were cultured in 150 mm cell culture dishes up to 80–90% of confluency. For chromatin fragmentation, extracts were incubated with 2 µl Micrococcal Nuclease (Cell Signaling, 10011) and sonicated in a Bioruptor (Diagenode) for 2 cycles at high intensity. Extracts were incubated with 5 µg of anti-DHX9 (Abcam, ab26271) or anti-IgG (Cell Signaling, 2729) overnight at 4 °C. qPCR was performed on a 7900HT Fast Real-Time PCR System (Applied Biosystems) using primers and iTaq Universal SYBR Green Supermix (Biorad, 1725270). ChIP-qPCR data were analyzed using the percent input method. Primers are shown in Supplementary Table 6.

### MTT assay and drug combinations

Cells were seeded in 96-well cell culture plates at a density of 1.5–3 × 10^3^ cells per well. To analyze the sensitivity to SN-38, cells were treated with indicated concentrations of SN-38 or DMSO for 72 h before MTT assay (Roche, 11465007001), according to the manufacturer’s instructions. For the combination of SN-38 with DOX or YK-4-279 inhibitor, cells were pre-treated with 1 µg/ml of DOX or 75 µM of YK-4-279 for 24 h or 1 h, respectively. Then, cells were treated with indicated concentrations of SN-38 for 3 h, washed, and maintained in a drug-free medium for 48 h before the MTT assay. Absorbance was measured in a microplate reader (TECAN) at 565 nm. Survival curves and IC50 were determined using GraphPad PRISM version 9 (GraphPad Software Inc., CA, USA).

For SN-38 and ATRi combination, cells were treated with AZD6738 ATRi Ceralasertib for 24 h at indicated concentrations before treatment with SN-38. Cells were maintained in a drug-containing medium during 72 h before MTT assay. Absorbance was measured in a microplate reader (TECAN) at 565 nm. Synergy between SN-38 and ATRi was evaluated using SynergyFinder+ web application [29].

### Analysis of microarray, DRIP-seq and RNA-seq public data

Analysis of microarray data was performed using GEO2R (NCBI Gene Expression Omnibus), or R software (version 4.2.2) and “affy” package from Bioconductor. Expression data were normalized using the robust multi-array average (RMA) method. DRIP-seq data were analyzed using Galaxy web-based platform. Briefly, reads were mapped against hg19 human reference genome using Bowtie2. RNA-seq data were analyzed using Galaxy web-based platform. After quality control, reads were mapped against hg19 human reference genome using Bowtie2 tool. Data were visualized using UCSC genome browser [30]. Accession numbers are shown in Supplementary Table 8.

### Survival analysis

For the evaluation of the association between *DHX9* mRNA levels and EwS patient outcome, survival analysis was performed as previously described [31] in a cohort of 166 primary EwS tumors with available clinical annotations. Patients were grouped according to *DHX9* mRNA levels into: high (first quartile) and moderate-low (second to last quartile). *P*-value was determined by Mantel-Cox test using GraphPad PRISM version 9 (GraphPad Software Inc., CA, USA).

### Statistical analysis

Statistical analysis was performed using GraphPad PRISM version 9 (GraphPad Software Inc., CA, USA). *P*-value is indicated in the figures. Comparison tests are defined and shown in the figure legends. Similar variance was assumed between groups. Biological replicates were chosen based on previous studies, which indicate that this sample size provides enough power to detect the expected effect. Our results also show low variability across replicates, supporting the reliability of this approach. Additionally, using three replicates is a common practice in the field, balancing statistical robustness with practical feasibility. The number of animals in our PDX models was adjusted to the minimum required to ensure robust and reliable results while adhering to the principles of the Three Rs (Reduction, Refinement, and Replacement).

## Results

### EwS cell lines and tumors are highly sensitive to TOP1 poisons

**Fig. 1:**
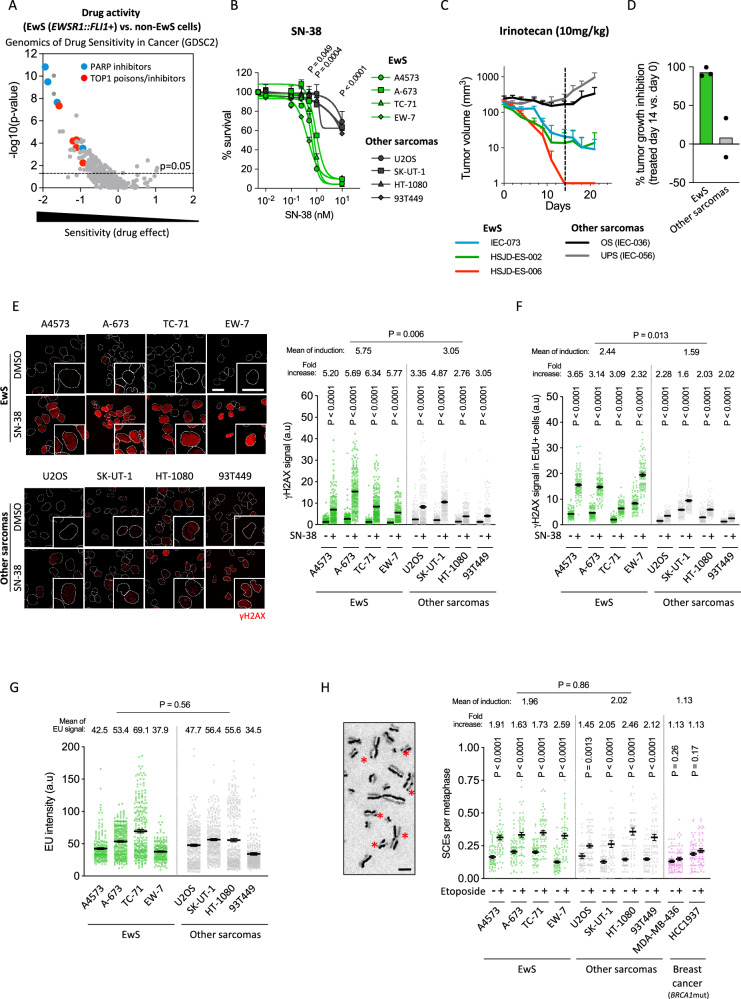
EwS cell lines and tumors are highly sensitive to TOP1 poisons. **A** Comparative analysis of drug activity between 15-17 EwS (carrying the *EWSR1::FLI1* fusion oncogene) and more than 600 non-EwS cell lines using the Genomics of Drug Sensitivity in Cancer website. Each dot is a tested drug. **B** Evaluation of SN-38 sensitivity between EwS (A4573, A-673, TC-71, and EW-7) and non-EwS cell lines (U2OS (osteosarcoma), SK-UT-1 (leiomyosarcoma), HT-1080 (fibrosarcoma), and 93T449 (liposarcoma)). After 72 h of treatment with indicated concentrations, cell viability was analyzed by MTT. Data represent the mean (+SEM) of the percentage of survival (relative to DMSO); *n* = 2 independent experiments. **C** Evaluation of tumor growth of PDX models (EwS: IEC-073, HSJD-ES-002, HSJD-ES-006; osteosarcoma, IEC-036; UPS, IEC-056) over 21 days following the beginning of irinotecan treatment. The dotted line indicates the end of treatment. Data represent the mean (+SEM) of tumor volume. **D** Evaluation of tumor growth inhibition of PDX models at the end of irinotecan treatment. Data represent the mean of the percentage of tumor growth inhibition. **E** Analysis of SN-38-induced DSBs comparing EwS (A4573, A-673, TC-71 and EW-7) and non-EwS cell lines (U2OS, SK-UT-1, HT-1080 and 93T449) by γH2AX IF. Cells were treated with 5 µM of SN-38 (30 min). *Left*, representative images. DAPI counterstain. Scale bar, 20 µm. *Right*, data represent the mean (±SEM) of nuclear γH2AX intensity; *n* ≥ 2 independent experiments (~75 cells were analyzed per replicate). **F** Similar to (**E**) in S-phase population (determined by EdU incorporation). Cells were incubated with 10 µM of EdU (20 min), washed and treated with 5 µM SN-38 (30 min). Data represent the mean (±SEM) of nuclear γH2AX intensity in EdU-positive cells (a total of ~100 cells were analyzed). **G** Determination of transcriptional rates between EwS and non-EwS cell lines by EU incorporation. Cells were incubated with 0.5 mM of EU for 45 min. Data represent the mean (±SEM) of nuclear EU intensity; *n* ≥ 2 independent experiments (~100 cells were analyzed per replicate). **H** Study of HR efficiency by SCEs assay in EwS, non-EwS, and *BRCA1*-mutated breast cancer cell lines (MDA-MB-436 and HCC-1937). Cells were treated with 2.5 µM etoposide for 30 min. *Left*, representative image. Scale bar, 5 µm. Asterisks indicate SCEs events. *Right*, data represent the mean (±SEM) of the frequency of SCEs per metaphase; *n* = 3 independent experiments (~20 metaphases were analyzed per replicate). *P*-value was determined by *t*-test.

To elucidate the molecular mechanisms underlying drug sensitivity in EwS, we interrogated the Genomics of Drug Sensitivity in Cancer database [32]. We compared the activity of more than 300 compounds on 15–17 EwS cell lines (carrying *EWSR1::FLI1* fusion oncogene) with respect to more than 600 non-EwS cells. Data indicated that EwS cell lines were significantly more sensitive to drugs commonly used as chemotherapeutic agents, such as PARP inhibitors and DNA damaging agents (Fig. 1A). Interestingly, we found a marked hypersensitivity of EwS cells to DNA topoisomerase 1 (TOP1) poisons and inhibitors, including irinotecan, topotecan and camptothecin (CPT) (Fig. 1A, Supplementary Fig. 1A), in agreement with previous reports [33]. To validate these observations, we compared the sensitivity of EwS (A4573, A-673, TC-71, EW-7) and non-EwS sarcoma (U2OS, SK-UT-1, HT-1080, 93T449) cell lines to SN-38, the active metabolite of irinotecan, which confirmed that EwS cells are significantly more sensitive than non-EwS cell lines (Fig. 1B).

Next, we studied irinotecan sensitivity in vivo using EwS (IEC-073, HSJD-ES-002, HSJD-ES-006) and non-EwS (IEC-036, osteosarcoma, OS; IEC-056, undifferentiated pleomorphic sarcoma, UPS) patient-derived xenografts (PDXs) models (Supplementary Table 1). Tumors were subcutaneously implanted and treated as previously described [34]. Evaluation of tumor volume during 21 days after the beginning of the treatment showed a higher response of EwS compared to non-EwS models (Fig. 1C), which is concordant with the potent activity of irinotecan in EwS PDXs [35]. For the analysis of tumor growth inhibition, we compared tumor volumes at the start (day 0) and end (day 14) of treatment. Data showed that growth inhibition was significantly higher in EwS PDX models (92.83%) compared to OS (33.67%) and UPS (−17.02%)(Fig. 1D). Indeed, 95% (19/20) of EwS tumors showed a complete response to irinotecan while most of OS and UPS tumors showed a partial response or stable disease (Supplementary Fig. 1B). Tumor response was also assessed by evaluating the treatment-induced necrosis at day 7, which was significantly higher in EwS PDXs (78.75%) compared to OS (40%) and UPS (46.5%) models (Supplementary Fig. 1C). Finally, evaluation of event-free survival showed that treated EwS PDX models survived more than 60 days without evidence of tumor growth, while OS and UPS animals were sacrificed upon achieving endpoint (tumor volume around 1200 mm^3^) at 32.5 and 26.5 days after the start of the treatment, respectively (Supplementary Fig. 1D).

Altogether, these results demonstrated that EwS is significantly more sensitive to TOP1 poisons than other tested sarcoma models, both in vitro and in vivo.

### TOP1 poisons cause more DNA damage in EwS than in other tumoral cells, independent on proliferation, transcription, and recombination differences

TOP1-poisoning-induced single-strand breaks are readily converted into double-strand breaks (DSBs), the most cytotoxic form of DNA damage, mainly through collision with the replication machinery during S-phase [36]. Consequently, we analyzed whether the hypersensitivity of EwS cell lines to TOP1 poisons could be associated with a stronger induction of DSBs. Indeed, evaluation of phospho-H2AX (Ser139)(hereafter γH2AX), a surrogate marker of DSBs [37], indicated that SN-38 treatment induced higher levels of DSBs in EwS in comparison to non-EwS sarcoma cells (Fig. 1E). Importantly, the expression levels of *TOP1*, *TDP1*, or single-strand break repair factors are similar between EwS and non-EwS cells (Supplementary Fig. 1E). Genotoxic agents present different mechanisms for DSB induction; nevertheless, there are common cellular factors that modulate the formation of these lesions, such as replication or transcription. Therefore, differences in the activity of these processes may result in variations in DSB induction and drug cytotoxicity. To assess potential differences in proliferation, we first measured the percentage of cells in S-phase by flow cytometry, which yielded no significant differences between EwS and non-EwS cells (Supplementary Fig. 1F). Similarly, proliferation rates were analyzed by Ki-67 immunocytochemistry (ICC) in paraffin-embedded cell pellets showing no differences in Ki-67-positive cells between both groups (Supplementary Fig. 1G). MRC-5, a non-tumoral fibroblast cell line with low proliferation rate, was included as a control (Supplementary Fig. 1G). To further confirm that increased induction of DNA damage in EwS cells did not depend on differences in cell cycle, we specifically analyzed γH2AX levels in S-phase cells, determined by the incorporation of thymidine analog 5-ethynyl-2′-deoxyuridine (EdU). Interestingly, although EwS and non-EwS cell lines showed similar levels of EdU incorporation, indicating no differences in replication (Supplementary Fig. 1H), the induction of DSBs by SN-38 was significantly higher in EwS cells (Fig. 1F).

Next, we evaluated global transcription by detecting 5-ethynyluridine (EU), an analog of uridine incorporated into the nascent RNA. Results showed no significant differences in EU incorporation between EwS and non-EwS cells (Fig. 1G, Supplementary Fig. 1I). In addition, we analyzed the phosphorylation of the C-terminal domain of RNA polymerase II at serine 2 (hereafter RNAPII-Ser2p), showing similar staining between EwS and non-EwS cells (Supplementary Fig. 1J). These results suggest that EwS cells exhibit similar levels of proliferation, DNA replication, and transcription to non-EwS cells.

Finally, we reasoned that reduced HR efficiency, previously attributed to EwS cells, could cause DSB accumulation upon treatment with DNA-damaging agents. To evaluate that, we analyzed sister chromatid exchanges (SCEs), a well-established hallmark of HR, upon DSB induction with a low dose of etoposide. However, no significant differences were observed between EwS and non-EwS cells (Fig. 1H). Importantly, in all cases, induction of SCEs was markedly higher compared to *BRCA1*-mutated breast cancer cell lines, used as positive controls (Fig. 1H). In summary, these findings indicate that SN-38 induced higher levels of DNA damage in EwS than in non-EwS cells, which is not due to increased proliferation, DNA replication, or transcription, nor to reduced HR efficiency.

### EWS::FLI1 impairs drug-induced R-loop resolution promoting genome instability and cytotoxicity

**Fig. 2:**
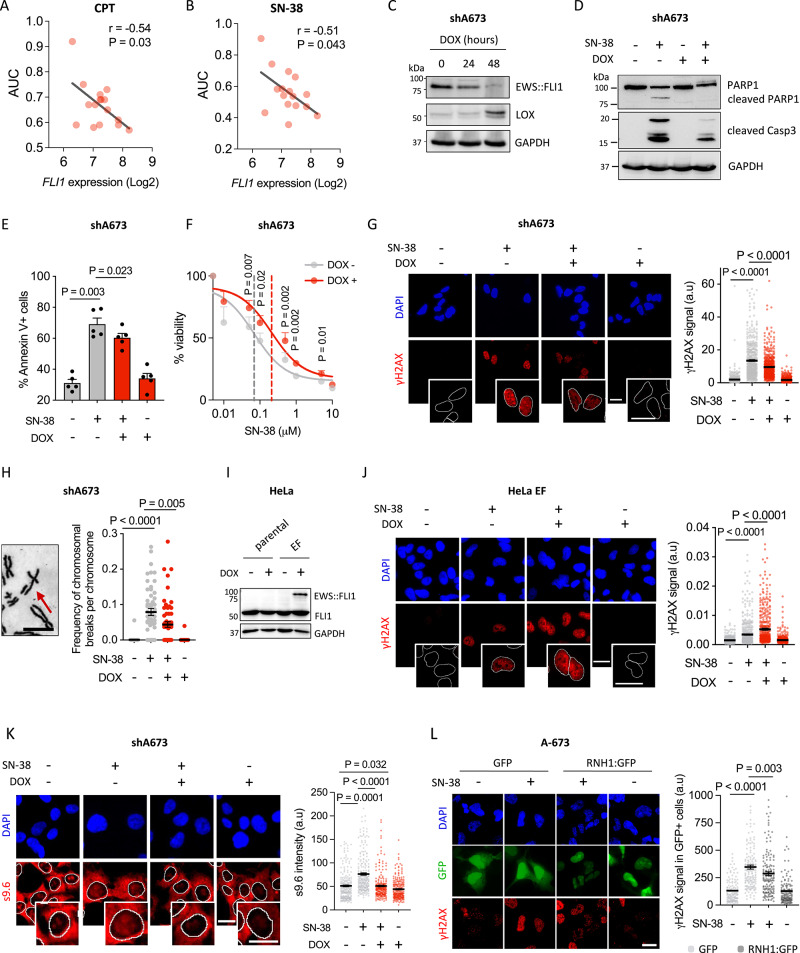
EWS::FLI1 impairs drug-induced R-loop resolution promoting genome instability and cytotoxicity. **A** Evaluation of the association between CPT and **B** SN-38 activity (AUC), and *EWSR1::FLI1* expression levels in EwS cell lines (*n* = 16). *FLI1* gene was used as a surrogate marker of *EWSR1::FLI1*, since *FLI1* is not expressed in EwS cells. Lineal correlation was determined by Pearson correlation coefficient. **C** EWS::FLI1 and LOX protein levels by WB in shA673 cells after DOX incubation. Loading control: GAPDH. Molecular weight in kDa. **D** SN-38-induced apoptosis upon *EWSR1::FLI1* knockdown by PARP1 and CASP3 WB. Cells were pre-incubated with DOX for 24 h before 5 µM SN-38 treatment (3 h). After washout, cells were cultured in drug-DOX-free medium for 24 h. Loading control: GAPDH. Molecular weight in kDa. **E** Similar to (**D**) by AnnexinV FACS. Data represent the mean (+SEM) of the percentage of AnnexinV-positive cells, *n* = 5 independent experiments. **F** Similar to (**D**) by MTT assay. Cells were treated with indicated concentrations of SN-38 (3 h). After treatment, cells were cultured in drug-DOX-free medium for 48 h. Data represent the mean (+SEM) of the percentage of survival (relative to DMSO), *n* = 5 independent experiments. Dotted lines indicate IC50. **G** SN-38-induced γH2AX upon *EWSR1::FLI1* knockdown by IF. Cells were pre-incubated with DOX for 24 h and treated with 5 µM SN-38 (30 min). *Left*, representative images. DAPI counterstain. Scale bar, 20 µm. *Right*, data represent the mean (±SEM) of nuclear γH2AX intensity, *n* = 5 independent experiments (~100 cells were analyzed per replicate). **H** SN-38-induced chromosomal breaks upon *EWSR1::FLI1* downregulation. After DOX incubation, cells were treated with 2.5 µM SN-38 (30 min). *Left*, representative image. Scale bar, 10 µm. The arrow indicates a chromosomal break. *Right*, data represent the mean (±SEM) of the frequency of chromosomal breaks per chromosome, *n* = 3 independent experiments (20 metaphases were analyzed per replicate). **I** EWS::FLI1 protein levels in HeLa EF cells after 72 h of DOX incubation. Loading control: GAPDH. Molecular weight in kDa. **J** SN-38-induced γH2AX upon *EWSR1::FLI1* overexpression by IF. HeLa EF cells were pre-incubated with DOX for 72 h and treated with 5 µM SN-38 (30 min). Other details as in (**G**), *n* = 3 independent experiments (~100 cells were analyzed per replicate). **K** SN-38-induced R-loops upon *EWSR1::FLI1* knockdown by s9.6 IF. Cells were pre-incubated with DOX and treated with 5 µM SN-38 (30 min). *Left*, representative images. DAPI counterstain. Scale bar, 20 µm. *Right*, data represent the mean (±SEM) of nuclear s9.6 intensity, *n* = 3 independent experiments (~75 cells were analyzed per replicate). **L** Effect of RNH1 overexpression on SN-38-induced DSBs. Cells were transfected with RNH1:GFP or control plasmids 24 h before treatment with 5 µM SN-38 (30 min). *Left*, representative images. DAPI counterstain. Scale bar, 20 µm. *Right*, data represent the mean (±SEM) of nuclear γH2AX intensity in GFP-positive cells, *n* = 3 independent experiments (~50 cells were analyzed per replicate). *P*-value was determined by *t*-test.

Since TET::ETS fusions are the main drivers of EwS, we next studied the contribution of EWS::FLI1 to the hypersensitivity to TOP1 poisons of EwS cells. Analysis of public gene expression and drug activity data showed a significant negative correlation between *EWSR1::FLI1* levels and the area under the curve (AUC) for CPT and SN-38 treatments, suggesting that EWS::FLI1 promotes EwS sensitivity to TOP1 poisons (Fig. 2A, B). To confirm these findings, we employed the pre-established A-673/TR/shEF model (hereafter shA673), carrying a doxycycline (DOX) inducible shRNA against *EWSR1::FLI1* [38]. shA673 cells exposed to DOX exhibited decreased EWS::FLI1 both at mRNA and protein levels (Fig. 2C, Supplementary Fig. 2A), leading to an upregulation of *LOX* (an EWS::FLI1 repressed target) [39], and a gradual accumulation of cells in G1 phase of the cell cycle, as previously described (Supplementary Fig. 2A, B). Given that changes in cell proliferation could affect drug activity, experiments were carried out at 24 h of DOX incubation. At this time point, no significant differences were observed in cell cycle nor replication activity, as measured by EdU incorporation (Supplementary Fig. 2B, C). To analyze the contribution of EWS::FLI1 to SN-38 cytotoxicity, shA673 cells were incubated with DOX for 24 h before treatment with SN-38. Notably, mild depletion of EWS::FLI1 significantly reduced SN-38-associated cleavage of PARP1 and Casp3, suggesting that EWS::FLI1 increases drug-induced apoptosis (Fig. 2D). In agreement, *EWSR1::FLI1* knockdown significantly reduced the percentage of drug-induced AnnexinV-positive cells (Fig. 2E). Finally, in accordance with these results, *EWSR1::FLI1* silencing significantly increased cell survival to SN-38 (IC50 from 0.07 to 0.2 µM)(Fig. 2F), demonstrating that EWS::FLI1 promotes EwS sensitivity to TOP1 poisons (Fig. 2A, B).

Next, to elucidate whether EWS::FLI1 modulates the induction of DSBs by TOP1 poisons, we analyzed γH2AX levels upon DOX incubation. Strikingly, downregulation of *EWSR1::FLI1* significantly reduced the levels of SN-38 and CPT-induced DNA damage (Fig. 2G, Supplementary Fig. 2D). In agreement with our previous result (Fig. 1E), pannuclear γH2AX staining suggested a major enrichment of damaged S-phase cells. Importantly, this reduction was independent of changes in the cell cycle, nor to differences in SN-38-induced TOP1 degradation, or TDP1 or single-strand break repair factors levels (Supplementary Fig. 2E, G). Since γH2AX is a surrogate marker of DSBs, we directly measured chromosomal breaks in spread preparations. In accordance with our previous results, we observed that pre-treatment with DOX significantly reduced drug-induced chromosomal breaks (Fig. 2H). To further confirm the role of EWS::FLI1 in promoting TOP1-poisoning-associated DNA damage, we used a pre-stablished HeLa model which expresses the *EWSR1::FLI1* oncogene under the control of a DOX-inducible promoter (Fig. 2I) [24]. Interestingly, overexpression of *EWSR1::FLI1* resulted in a significant increase of SN-38-induced DSBs (Fig. 2J).

To clarify the mechanism behind the EWS::FLI1-dependent induction of DSBs by TOP1 poisons, we first analyzed the potential modulation of HR efficiency by EWS::FLI1. As expected from our previous result showing no differences in HR activity between EwS and non-EwS cells (Fig. 1H), we observed that *EWSR1::FLI1* silencing at the depletion times used did not alter HR efficiency, measured by SCEs and DRGFP reporter plasmid assays (Supplementary Fig. 2H, I).

Next, considering that TOP1 poisoning was previously associated with a rapid accumulation of R-loops [40], a prominent source of DNA damage and genome instability, we tested whether EWS::FLI1 could affect the metabolism of drug-induced R-loops. As previously described, TOP1 poisoning caused a significant decrease in transcription, measured by EU incorporation (Supplementary Fig. 2J, K). To analyze whether TOP1 poisoning caused R-loops accumulation in EwS cells, we measured R-loops levels by immunofluorescence microscopy using the antibody s9.6. Interestingly, SN-38 treatment induced a significant accumulation of nuclear R-loops (Supplementary Fig. 2L). Notably, s9.6 signal was markedly reduced by RNH1 treatment, indicating the specificity of the antibody (Supplementary Fig. 2L). More importantly, downregulation of *EWSR1::FLI1* in shA673 cells significantly reduced the levels of SN-38-induced nuclear R-loops (Fig. 2K), suggesting that EWS::FLI1 plays a role in the metabolism of drug-induced R-loops. More importantly, R-loop depletion by overexpression of RNH1 in A-673 cells significantly reduced SN-38-induced DSBs (Fig. 2L, Supplementary Fig. 2M), demonstrating the contribution of SN-38-induced R-loops to the generation of genome instability in EwS cells.

### Loss of EWS::FLI1-DHX9 interaction prevents SN-38-induced R-loops accumulation and genome instability

**Fig. 3:**
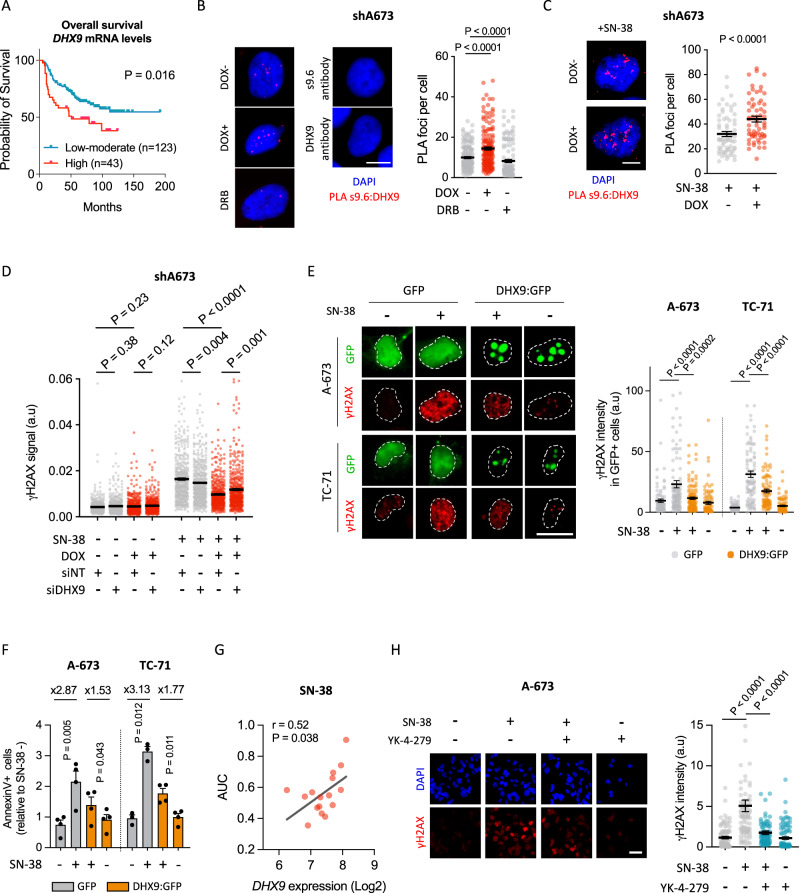
Loss of EWS::FLI1-DHX9 interaction alleviates R-loops accumulation promoting drug resistance. **A** Kaplan–Meier showing overall survival of 166 EwS patient samples stratified by *DHX9* mRNA levels. Statistical significance was determined by the Mantel–Cox test. **B** Determination of R-loops-DHX9 interactions by PLA in shA673 cells upon DOX incubation, and **C** DOX incubation and 5 µM SN-38 treatment (30 min). *Left*, representative images (red, PLA foci; blue, DAPI counterstain). Scale bar, 20 µm. *Right*, data represent the mean (±SEM) of PLA foci, *n* = 3 independent experiments (40 (**B**) and 20 (**C**) cells were analyzed per replicate). **D** Evaluation of the effect of *DHX9* downregulation on SN-38-induced DSBs upon EWS::FLI1 depletion by γH2AX IF. After 48 h of transfection with indicated siRNAs and/or 24 h of incubation with DOX, shA673 cells were treated with 5 µM SN-38 (30 min). Data represent the mean (±SEM) of nuclear γH2AX signal; *n* = 3 independent experiments (150 cells were analyzed per replicate). **E** Evaluation of the effect of *DHX9* overexpression on SN-38-induced DSBs by γH2AX IF. A-673 and TC-71 cells overexpressing DHX9:GFP or control plasmids were treated with 5 µM SN-38 (30 min). *Left*, representative images. DAPI counterstain. Scale bar, 20 µm. *Right*, data represent the mean (±SEM) of nuclear γH2AX signal of GFP-positive cells, n ≥ 3 independent experiments (30 cells were analyzed per replicate). **F** Effect of *DHX9* overexpression on SN-38-induced apoptosis by AnnexinV FACS. Cells overexpressing DHX9:GFP or control plasmids were treated with 5 µM SN-38 (3 h). After washout, cells were maintained in drug-free medium for 24 h. Data represent the mean (+SEM) of the percentage of AnnexinV-positive between GFP-positive cells (relative to SN-38-), *n* ≥ 3 independent experiments. **G** Evaluation of the association between SN-38 activity (AUC) and *DHX9* expression levels in EwS cell lines (*n* = 16). Lineal correlation was determined by the Pearson correlation coefficient. **H** Analysis of the effect of YK-4-279 on SN-38-induced DSBs by γH2AX IF. A-673 cells were pre-incubated with 75 µM YK-4-279 (1.5 h) and treated with 5 µM SN-38 (30 min). *Left*, representative images. DAPI counterstain. Scale bar, 50 µm. *Right*, data represent the mean (±SEM) of nuclear γH2AX intensity, *n* = 3 independent experiments (30 cells were analyzed per replicate). *P*-value was determined by *t*-test.

To evaluate the mechanism behind EWS::FLI1-dependent R-loop accumulation in response to SN-38, on the one hand, we focused on the potential role of EWS::FLI1 in the transcriptional regulation of R-loop-interacting factors. To address that, we crossed four datasets comprising: EWS::FLI1 transcriptionally regulated genes (A673/TR/shEF; 36 h DOX treatment) [41], differentially expressed genes between EwS and non-EwS sarcoma cell lines [42], and two R-loop interactomes [17, 43] (Supplementary Fig. 3A). Surprisingly, we didn’t find common shared genes between all datasets, suggesting that altered metabolism of R-loops in EwS cells may not be attributed to EWS::FLI1-dependent dysregulation of R-loops processing factors (Supplementary Fig. 3A). On the other hand, we investigated EWS::FLI1-interacting proteins hypothesizing that EWS::FLI1 could affect the role of these factors in R-loops metabolism. Evaluation of a published EWS::FLI1 [44], and R-loop interactomes highlighted 21 factors that interact both with EWS::FLI1 and R-loops (Supplementary Fig. 3B). Among them, we focused on DHX9 due to its previously described activity in the resolution of TOP1-poisoning-induced R-loops [17], and its crucial role in EwS tumorigenesis [23]. Indeed, DHX9 is highly expressed in EwS cell lines and tumors with respect to other entities (Supplementary Fig. 3C, D). In agreement, evaluation of DHX9 in a representative cohort of 205 EwS primary samples by IHC, showed that more than 65% of samples present high levels of DHX9 (Supplementary Fig. 3E). More importantly, exploration of available datasets comprising clinically annotated EwS transcriptomes revealed a significant association between high *DHX9* expression and reduced overall survival (Fig. 3A).

Then, we investigated whether EWS::FLI1 disrupts the binding of DHX9 to the R-loops by proximity ligation assay (PLA) using s9.6 and anti-DHX9 antibodies. Results indicated that downregulation of *EWSR1::FLI1* significantly increased the interaction between DHX9 and both endogenous and SN-38-induced R-loops (Fig. 3B, C). Importantly, knockdown of *EWSR1::FLI1* did not alter the levels of DHX9 (Supplementary Fig. 3F). Strikingly, *DHX9* knockdown increased γH2AX levels in EWS::FLI1-depleted cells, suggesting that DHX9 availability plays a key role in suppressing DNA damage accumulation following *EWSR1::FLI1* knockdown (Fig. 3D, Supplementary Fig. 3G). In agreement, overexpression of DHX9 in A-673 and TC-71 cells was associated with a significant reduction of SN-38-induced DSBs and cytotoxicity (Fig. 3E, F, Supplementary Fig. 3H). Additionally, we found a positive and significant correlation between *DHX9* expression levels and the AUC for SN-38 treatment in a set of 16 EwS cell lines, suggesting that DHX9 promotes drug resistance (Fig. 3G). To confirm that EWS::FLI1-DHX9 interaction is a source of drug-associated genome instability, we used the small molecule YK-4-279, which, among other effects, has shown efficacy in blocking EWS::FLI1-DHX9 binding [45]. Strikingly, pre-treatment of A-673 cells with YK-4-279 significantly reduced SN-38-induced γH2AX and chromosomal breaks promoting drug resistance (Fig. 3H, Supplementary Fig. 3I, J).

Collectively, these findings indicate that EWS::FLI1-DHX9 interaction prevents SN-38-induced R-loops resolution, promoting its accumulation and the induction of genome instability, which has a potentially significant prognostic relevance.

### SN-38-induced R-loops strengthen replication stress in EwS

**Fig. 4:**
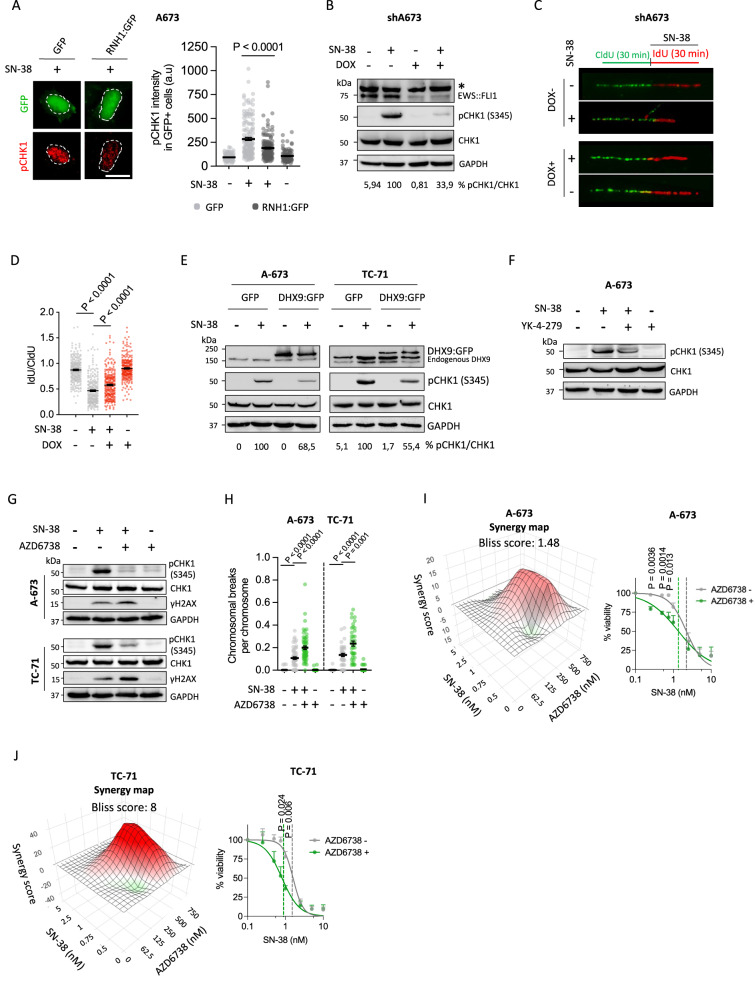
EWS::FLI1-DHX9 associated R-loop accumulation is a source of replication stress. **A** Analysis of the effect of RNH1 overexpression in SN-38-induced replication stress by pCHK1 (Ser345) IF. Cells were transfected with RNH1:GFP or control plasmids 24 h before 5 µM SN-38 treatment (30 min). *Left*, representative images. DAPI counterstain. Scale bar, 20 µm. *Right*, data represent the mean (±SEM) of pCHK1 intensity in GFP-positive cells, *n* ≥ 2 independent experiments (30 cells were analyzed per replicate). **B** Effect of *EWSR1::FLI1* knockdown in SN-38-induced replication stress by pCHK1 (Ser345) WB. shA673 cells were pre-incubated with DOX and treated with 5 µM SN-38 (30 min). Loading control: GAPDH. Molecular weight in kDa. Asterisk indicates non-specific bands. *Bottom*, quantification of CHK1 phosphorylation (pCHK1/CHK1 band signal). Data represent the mean of 2 independent experiments (relative to SN-38). **C** Similar to (**B**), by DNA fiber assay. Cells were incubated with CidU (30 min) and IdU + 5 µM SN-38 (30 min). **D** Quantification of (**C**). Data represent the mean (±SEM) of IdU/CidU fiber length ratio, *n* = 2 independent experiments (more than 250 fibers were analyzed per condition). **E** Effect of DHX9 overexpression on SN-38-induced replication stress by pCHK1 (Ser345) WB. A-673 and TC-71 cells overexpressing DHX9:GFP or control plasmids were treated with 5 µM SN-38 (30 min). *Bottom*, quantification of CHK1 phosphorylation (pCHK1/CHK1 band signal). Data are the mean of 2 independent experiments (relative to SN-38 GFP). Other details as in (**B**). **F** Effect of YK-4-279 treatment on SN-38-induced replication stress by pCHK1 (Ser345) WB. Cells were pre-incubated with 75 µM YK-4-279 (1.5 h) and treated with 5 µM SN-38 (30 min). Other details as in (**B**). **G** Evaluation of the effect of ATR inhibition in SN-38-induced pCHK1 and γH2AX by WB. A-673 and TC-71 cells were pre-incubated with 10 µM AZD6738 for 24 h and treated with 5 µM SN-38 (30 min). Other details as in (**B**). **H** Analysis of the effect of ATR inhibition in SN-38-induced chromosomal breaks. Cells were pre-incubated with 10 µM AZD6738 for 24 h and treated with 2.5 µM SN-38 (A-673) or 1.25 µM SN-38 (TC-71) for 30 min. Data represent the mean (±SEM) of the frequency of chromosomal breaks per chromosome, *n* ≥ 2 independent experiments (20 metaphases were analyzed per replicate). Statistical significance was determined by *t*-test. **I** *Left*, synergy plot representing synergistic effect of AZD6738 and SN-38 combination in A-673 cells. *Right*, effect of ATR inhibition in cell survival upon SN-38 exposure by MTT assay. Cells were incubated with AZD6738 (250 nM, 24 h) previous to the treatment with indicated concentrations of SN-38 (72 h). Data represent the mean (+SEM) of the percentage of survival, *n* = 3 independent experiments. Dotted lines indicate IC50. **J** Similar to (**I**) in TC-71 cell line. Statistical significance was determined by *t*-test.

Replication stress is an important source of R-loop-associated genome instability in cycling cells [12]. Notably, overexpression of RNH1 in A-673 cells significantly reduced the levels of SN-38-induced phospho-CHK1 (Ser345)(pCHK1), a replication stress marker, indicating that R-loops may mediate replication stress induced by TOP1 poisons (Fig. 4A). Additionally, in agreement with the observed decrease of drug-induced R-loops (Fig. 2K), knockdown of *EWSR1::FLI1* strongly reduced SN-38 and CPT-induced pCHK1 (Fig. 4B, Supplementary Fig. 4A). Similarly, *EWSR1::FLI1* silencing was associated with a significant decrease of phospho-RPA32 (Ser4 and Ser8)(pRPA32 s4 + s8), a different replication stress marker (Supplementary Fig. 4B). To confirm the origin of replication stress we directly monitored DNA replication using the DNA fiber technique measuring incorporation of CldU, followed by IdU, in the presence of SN-38. As previously shown, SN-38 hindered replication progression in shA673 cells (Fig. 4C, D). Strikingly, EWS::FLI1 depletion partially prevented this defect (Fig. 4C, D). Finally, the comparison of the levels of drug-associated replication stress between EwS and non-EwS cells indicated that EwS cells accumulate higher levels of pCHK1 than other sarcomas upon SN-38 exposure (Supplementary Fig. 4C).

To evaluate whether EWS::FLI1 promotes replication stress through the interaction with DHX9, we first measured pCHK1 levels in A-673 and TC-71 upon DHX9 overexpression. Notably, high levels of DHX9 reduced pCHK1 levels induced by SN-38 (Fig. 4E). Indeed, pre-incubation of A-673 cells with YK-4-279 inhibitor also reduced SN-38-associated pCHK1 levels (Fig. 4F), suggesting that EWS::FLI1-DHX9 interaction promotes replication stress in EwS cells upon TOP1 poisoning. Taken together, these data indicate that SN-38 induces high levels of replication stress in EwS cells through the accumulation of R-loops.

Given that SN-38 induces R-loop-dependent replication stress in EwS cells, we investigated whether targeting the replication stress response could enhance SN-38 cytotoxicity. Then, we evaluated the combination of TOP1 poisons and ataxia telangiectasia mutated and rad3 related (ATR) inhibitors in vitro. ATR is a key kinase activated in response to replication stress that blocks replication fork progression to prevent the generation of DNA damage [46]. We employed the ATR inhibitor AZD6738 (Ceralasetib), which efficiently blocked ATR signaling pathway, reducing SN-38-induced pCHK1 levels (Fig. 4G). Interestingly, preincubation of A-673 and TC-71 cell lines with AZD6738 increased SN-38-induced DNA damage measured by γH2AX (Fig. 4G). Since ATR is one of the kinases that phosphorylates H2AX [47], we considered that we might be underestimating the effect of inhibiting ATR and directly measured DNA damage by scoring chromosomal breaks in spread preparations. In accordance with our previous result, ATR inhibition increased the levels of SN-38-induced chromosomal breaks in A-673 and TC-71 cells (Fig. 4H), indicating that blockage of replication stress signaling could enhance SN-38-induced genome instability in EwS cells.

Finally, we analyzed the potential synergistic effects of ATR inhibition and TOP1 poisoning. To this end, cells were pretreated with AZD6738 for 24 h before SN-38 treatment. Results indicated a synergistic effect (positive Bliss score) between both drugs at nanomolar concentrations (Fig. 4I, J, Supplementary Fig. 4D). More importantly, treatment with AZD6738 at a concentration of 250 nM—the concentration showing the highest synergistic effect- markedly reduced the IC50 of SN-38 in A-673 and TC-71 cells (Fig. 4I, J, Supplementary Fig. 4E). Taken together, these results demonstrate that ATR inhibition enhances EwS sensitivity to SN-38.

### EWS::FLI1 kidnaps DHX9 into the RNAPII transcriptional complex promoting R-loops formation

**Fig. 5:**
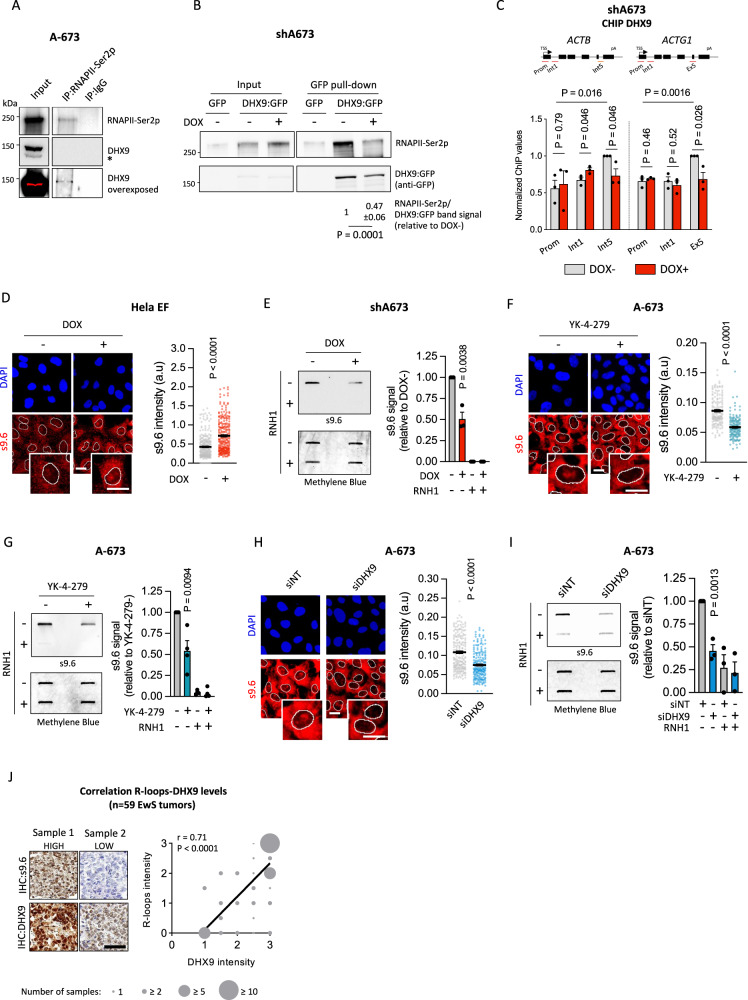
EWS::FLI1 kidnaps DHX9 into the RNAPII transcriptional complex promoting R-loops formation. **A** Analysis of the interaction between DHX9 and RNAPII-Ser2p by RNAPII-Ser2p pull-down. Molecular weight in kDa. Asterisk indicates non-specific bands. **B** Evaluation of the effect of *EWSR1::FLI1* knockdown in DHX9-RNAPII-Ser2p interaction. shA673 cells overexpressing DHX9:GFP or control plasmids were incubated with DOX for 24 h previous to GFP pull-down. Representative immunoblots. Molecular weight in kDa. Data represent the mean (±SD) of the RNAPII-Ser2p band signal, normalized to DHX9:GFP pull-down (relative to DOX-); *n* = 3 independent experiments. **C** Evaluation of chromatin distribution of DHX9 by ChIP upon *EWSR1::FLI1* knockdown. shA673 cells were treated with DOX for 24 h. *Upper,* diagram of *ACTB* and *ACTG1* genes; and primers used for qPCR (red lines). *Bottom*, data represent the mean (+SEM) of DHX9 ChIP signal (normalized to DOX- signal at 3′); *n* = 3 independent experiments. **D** Evaluation of the effect of *EWSR1::FLI1* overexpression on R-loops levels by s9.6 IF. HeLa EF cells were incubated with DOX for 72 h. *Left*, representative images. DAPI counterstain. Scale bar, 20 µm. *Right*, data represent the mean (±SEM) of nuclear R-loops; *n* = 5 independent experiments (50 cells were analyzed per replicate). **E** Evaluation of the effect of *EWSR1::FLI1* downregulation on R-loops levels by slot blot assay. shA673 cells were incubated with DOX for 24 h. *Left*, representative immunoblots. Loading control: methylene blue staining. *Right*, data represent the mean (+SEM) of s9.6 signal (normalized to methylene blue and relative to DOX-), *n* = 4 independent experiments. **F** Similar to (**D**) in A-673 cells after treatment with 25 µM YK-4-279 for 4 h; *n* = 3 independent experiments. **G** Similar to (**E**) in A-673 cells after treatment with 75 µM YK-4-279 for 1.5 h; *n* = 3 independent experiments. **H** Similar to (**D**) in A-673 cells 48 h after transfection with indicated siRNAs; *n* = 4 independent experiments. **I** Similar to (**E**) in A-673 cells 48 h after transfection with indicated siRNAs; *n* = 3 independent experiments. Statistical significance was determined by t-test. **J** Analysis of the correlation between R-loops and DHX9 protein levels in a cohort of 59 EwS tumors. Scale bar, 50 µm. Correlation was determined by the Pearson correlation coefficient.

Based on the role of DHX9 in facilitating EWS::FLI1 transcriptional activity and the observed interaction between EWS::FLI1 and phosphorylated RNAPII [48], we speculated that EWS::FLI1 could modulate DHX9 interaction with the RNAPII transcription complex, affecting R-loops processing. First, we confirmed by PLA the interaction between EWS::FLI1 and elongating RNAPII (RNAPII-Ser2p)(Supplementary Fig. 5A). Notably, RNAPII-Ser2p co-precipitated DHX9 (Fig. 5A), confirming the interaction between both proteins. More importantly, knockdown of *EWSR1::FLI1* significantly reduced DHX9-RNAPII-Ser2p physical interaction, demonstrating that EWS::FLI1 increases DHX9 association to elongating RNAPII (Fig. 5B). To confirm these results, we carried out a chromatin immunoprecipitation assay (ChIP) against DHX9. Interestingly, we observed a significant enrichment of DHX9 signal at 3′ of two constitutive genes with respect to the promoters (Fig. 5C), in agreement with RNAPII-Ser2p distribution [49]. More interestingly, the knockdown of *EWSR1::FLI1* resulted in a significant reduction of DHX9 signal at this position (Fig. 5C). Considering that splicing alterations can also influence the association of DHX9 with RNAPII [19], we tested splicing proficiency. Notably, knockdown of *EWSR1::FLI1* changed exon-intron ratios of *ACTB* and *ACTG1* genes, suggesting a splicing alteration (Supplementary Fig. 5B). Altogether, these results indicate that EWS::FLI1 increases DHX9 interaction with the elongating form of RNAPII.

EwS cells are characterized by an accumulation of endogenous R-loops [10]. In fact, analysis of R-loop levels by s9.6 IHC in a tissue microarray including several sarcoma subtypes, corroborated that EwS samples accumulate higher levels of R-loops than other sarcomas (Supplementary Fig. 5C). Loss of s9.6 signal after in vitro treatment with RNH1, but not with RNAse A, indicates the specificity of the antibody (Supplementary Fig. 5D). Notably, overexpression of *EWSR1::FLI1* in the Hela EF model resulted in a significant increase of R-loops, measured by s9.6 IF (Fig. 5D). Moreover, s9.6 IF and ICC showed that *EWSR1::FLI1* silencing in shA673 cells significantly reduced R-loops levels (Fig. 2K, Supplementary Fig. 5E). These results were confirmed by slot blot assay (Fig. 5E). In vitro treatment with RNH1 was used as a control of s9.6 specificity (Fig. 5E, Supplementary Fig. 5F). Importantly, R-loops accumulation in EwS cells has been previously attribute to the dysregulation of cellular transcription by EWS::FLI1 [10], nonetheless, incubation of shA673 cells with DOX for 24 h did not significantly affect transcription rates, measured by EU incorporation and RNAPII-Ser2p levels (Supplementary Fig. 5G, H). As a control, shA673 cells were treated with DRB, that specifically blocks RNAPII elongation, reducing EU incorporation and RNAPII-Ser2p levels (Supplementary Fig. 5I, J). These data suggest that EWS::FLI1 could promote R-loops formation independently of a global increase in transcription. Indeed, re-analysis of public DRIP-seq and RNA-seq data showed that, in non-EWS::FLI1 transcriptional regulated genes *ACTB* and *ACTG1*, EwS cells displayed higher levels of R-loops than IMR-90 cell line, while no differences were observed in gene expression (Supplementary Fig. 5K, L). Considering that the binding between DHX9 and the elongating RNAPII promotes R-loops formation [19], we explored whether this interaction could be a source of R-loops accumulation in EwS cells. Importantly, treatment with YK-4-279 inhibitor resulted in a significant decrease of R-loops, indicating that EWS::FLI1-DHX9 interaction increases R-loops levels in EwS cells (Fig. 5F, G). Remarkedly, this effect is not due to a higher R-loops resolution, as *DHX9* downregulation also led to a reduction of R-loops levels (Fig. 5H, I). Importantly, YK-4-279 treatment or *DHX9* knockdown did not affect cellular transcription (Supplementary Fig. 5M, N). Finally, we evaluated the relationship between DHX9 and R-loop levels in a cohort of 59 EwS primary tumors. Results indicated a significant positive correlation (*r* = 0.71, *P* < 0.0001) (Fig. 5J). Altogether, these results indicate that EWS::FLI1 increases DHX9 interaction with the elongating form of RNAPII, favoring R-loop formation in the absence of DNA damage.

## Discussion

Therapeutical management of EwS, an aggressive bone and soft-tissue cancer, remains based on chemotherapy, radiotherapy, and surgery. It is associated with a high rate of treatment failure and tumor relapse, especially in patients with metastasis at diagnosis. In recent years, TOP1 poison irinotecan has been evaluated in clinical trials for recurrent or primary refractory EwS, in combination with alkylating agent temozolomide [7, 8], emerging as an effective and well-tolerated therapeutic regimen. In this study, we conducted a comprehensive analysis of the molecular mechanisms underlying EwS sensitivity to TOP1 poisons, considering that our findings could offer novel therapeutic opportunities for EwS treatment.

Our results demonstrated that EwS is highly sensitive to TOP1 poisons both in vitro and in vivo. In this study, we primarily used SN-38 as a TOP1 poison and confirmed some results with CPT. Given the high specificity of CPT derivatives used in clinical settings, we expect our findings to be generalizable to other TOP1 poisons. In this line, and considering that TET::ETS fusions are the driver of the disease, we evaluated the potential role of EWS::FLI1 in the modulation of SN-38 activity. Based on the intrinsic difficulty of studying EWS::FLI1 activity in EwS cell lines due to the essential role of EWS::FLI1 in cell survival and the lack of isogenic wild-type counterparts, we employed a well-established doxycycline-dependent shRNA targeting *EWSR1::FLI1* [38]. This isogenic system demonstrated that EWS::FLI1 fusion is responsible for SN-38 sensitivity, highlighting the particularity of EwS biology compared to other sarcomas. Bearing in mind that genotoxic chemotherapy is the backbone of current EwS treatment regimens, these results showed the relevant connection between *EWSR1::FLI1* expression and drug sensitivity for the improvement of EwS therapies.

Notably, EwS sensitivity to SN-38 was associated with a significant EWS::FLI1-dependent increase in DNA damage and genome instability. Intriguingly, SN-38-associated genome instability and sensitivity in EwS did not correlate with increased proliferation, DNA replication, or transcription compared with other less sensitive sarcoma models. Furthermore, we did not observe any significant differences in the efficiency of HR repair between EwS and non-EwS sarcomas, which is in agreement with the lack of the characteristic mutational signature of “HR-deficient” samples in EwS patients [50]. While EwS sensitivity to DNA damaging agents has been previously attributed to an EWS::FLI1-dependent deficiency of HR (10), our data suggest that SN-38 hypersensitivity is also caused by a mechanism not linked to a less efficient lesions repair, or to defects in the initiation of HR provoked by a disruption of the DHX9 activity in recruiting RPA and RAD51 to DNA damage [51]. In fact, in the conditions used in this study, mild depletion of EWS::FLI1 leads to drug resistant without affecting HR efficiency. Discrepancies with the model proposed by Gorthi et al. could be caused by the management of *EWSR1::FLI1* expression levels; nonetheless, both mechanisms are compatible.

Then we focused on R-loops due to the described accumulation of these hybrids upon TOP1 poisoning [40]. Strikingly, our data indicate that EwS cells accumulate drug-induced R-loops depending on EWS::FLI1, and that the enhancement of R-loop resolution by RNH1 overexpression resulted in a significant decrease in DNA damage. This result suggests that drug-induced R-loops mediate the high levels of genome instability induced by SN-38 in EwS cells.

Exploration of different databases suggested that EWS::FLI1 did not modulate the expression of R-loops processing factors. Nevertheless, we observed that EWS::FLI1 interacts with proteins involved in R-loop metabolism with clinical relevance for EwS outcome. Among them, we focused on DHX9, which has been previously implicated in the processing of TOP1 poison-associated R-loops. Our data showed that EWS::FLI1 and DHX9 interact with RNAPII during transcription elongation, in agreement with previous data indicating the important role of DHX9 for EWS::FLI1-dependent transcriptional regulation. Indeed, knockdown of *EWSR1::FLI1* reduced the interaction between DHX9 and RNAPII-Ser2p, favoring the binding of DHX9 to SN-38-induced R-loops. These results suggested that EWS::FLI1 could hijack DHX9, preventing R-loop processing and inducing sensitivity to SN-38. Indeed, our data demonstrate that DHX9 plays a role in the prevention of SN-38-induced DNA damage accumulation in EWS::FLI1-depleted cells. Importantly, overexpression of DHX9 resulted in a reduction of TOP1-poisoning-induced DNA damage and cytotoxicity, revealing that EWS::FLI1 phenocopies DHX9 depletion. We confirmed these results employing the small molecule YK-4-279, the only published compound that has shown efficacy in blocking EWS::FLI1-DHX9 binding [45]. Nevertheless, the use of YK-4-279 has some important limitations. First, it blocks several protein–protein interactions between multiple ETS proteins and binding partners. Additionally, it may block tubulin polymerization [52]. Moreover, as previously described [48, 53], we detected that EWS::FLI1 leads to splicing defects in EwS cells, which may also promote the interaction between DHX9 and RNAPII-Ser2p [19]. Little is known about the mechanism that could modulate the levels and/or activity of DHX9 in EwS. Exploration of two EwS public cohorts using cBioportal indicated that the mutational frequency of DHX9 in EwS patients is lower than 1% [54, 55]. However, elevated DHX9 levels could be associated with structural alterations affecting its locus (chr1q25). This is extremely interesting, considering that gain of this chromosomal arm occurs in about 18% of EwS patients and is associated with a poor prognosis [54, 55].

Non-physiological R-loops pose a threat to genome stability, especially during DNA replication. Accordingly, SN-38 exposure resulted in R-loop-associated replication stress in EwS cells. A key discovery of this study is that replication stress caused by SN-38 treatment is significantly dependent on EWS::FLI1 fusion and can be prevented by RNH1 overexpression. Indeed, EwS cells showed higher levels of drug-induced replication stress compared to non-EwS ones. These results agree with the pannuclear pattern of γ-H2AX staining observed in EwS cells upon SN-38 treatment, characteristic of DNA damage in the S-phase. Importantly, we demonstrated that high replication stress is promoted by defects in replication fork progression, which are dependent on the EWS::FLI1 fusion. This reveals that alterations in R-loops resolution in EwS cells contribute to its hypersensitivity to SN-38. It is important to note that R-loop formation and TOP1 poisoning have been linked to DNA breaks induced by DNA nicks and DSBs, which favor the hybridization between the RNA and the template strand [12]. Given the rapid increase of R-loop formation by SN-38 and the significant reduction in CHK1 phosphorylation induced by SN-38 following *EWSR1::FLI1* knockdown, we suggest that the transient stabilization of transcription-associated R-loops by TOP1 poisoning is the most likely explanation. In addition, the formation of DNA:RNA hybrids after DNA damage has been linked to a more efficient DNA repair, promoting both NHEJ and HR [56]. However, while EWS::FLI1 depletion reduced the levels of drug-induced R-loops, no differences were observed in HR efficiency, reinforcing that R-loops are transiently stabilized by TOP1 poisoning, leading to the formation of DSBs.

Based on that, we decided to evaluate the combination of TOP1 poisons and inhibitors of ATR, a key factor in the signaling of replication stress. Interestingly, inhibition of ATR led to increased DNA breakage and exacerbated SN-38 sensitivity, suggesting the combination of TOP1 poisons and ATR inhibitors (an approach previously explored with each as single-agent treatments [57]) as a rational and promising regimen for preclinical evaluation.

**Fig. 6:**
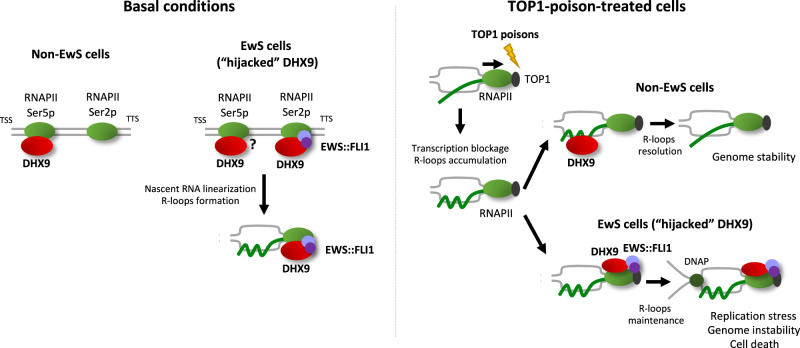
MODEL. Under basal conditions, EWS::FLI1 promotes the interaction between DHX9 and the elongating RNAPII leading to the formation of R-loops. In addition, TOP1 poisoning provokes the accumulation of R-loops through the blockage of RNAPII transcription. In non-EwS cells, DHX9 resolves drug-induced R-loops, preventing genome instability. In EwS cells, EWS::FLI1 “hijacks” DHX9 into the RNAPII transcriptional complex, reducing DHX9 recruitment to the R-loops and R-loops resolution. Replication machinery collapse with unresolved R-loops promoting replication stress, genome instability, and cell death.

EwS cells showed fusion-dependent interaction of DHX9 and the elongating form of RNAPII, supporting a model in which EWS::FLI1 promotes DHX9 association to RNAPII during transcription, similarly to the pattern promoted by defects on splicing [19]. In agreement with previous findings, showing that the binding between DHX9 and RNAPII-Ser2p is a source of R-loops formation, our results demonstrated that EWS::FLI1-DHX9 interaction contributes to the high levels of endogenous R-loops in EwS cells. Despite R-loops accumulation has been previously associated with increased cellular transcription driven by EWS::FLI1 [10], our results demonstrate that mild depletion of EWS::FLI1 reduced R-loops levels without affecting transcription. These findings support a mechanism -compatible with the one proposed by Gorthi and colleagues- in which EWS::FLI1 leads to the accumulation of R-loops by sequestering DHX9 into the RNAPII complex (Fig. 6). Importantly, we corroborated this connexion as high levels of DHX9 significantly correlated with high levels of R-loops in EwS patients. Nonetheless, further work is required to confirm this observation and to study the possible role of endogenous R-loops in EwS biology.

Overall, our results demonstrate that EWS::FLI1 modulates the activity of DHX9 in the metabolism of R-loops impeding the resolution of those induced by SN-38 (Fig. 6). This leads to heightened sensitivity of EwS cells to TOP1 poisons. Our research enhances the understanding of the mechanisms behind EwS cells’ sensitivity to genotoxic agents and could facilitate the development of new combination therapies in the future.

## Supplementary information

uncropped blots

Supplementary Figures

Supplementary Tables

## Source data

Source Data

## Data Availability

All data generated or analyzed during this study are included as a Source Data file or are available from the corresponding authors on reasonable request.
