# Supplementary figures and images for: EWS::FLI1-DHX9 interaction promotes Ewing sarcoma sensitivity to DNA topoisomerase 1 poisons by altering R-loop metabolism
Source: Oncogene. 2025 Jul 28;44(38):3537–52. doi: 10.1038/s41388-025-03496-9 (PMC12436182; doi:10.1038/s41388-025-03496-9)

2C

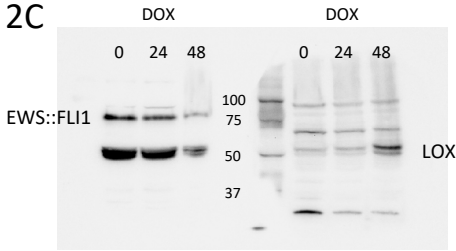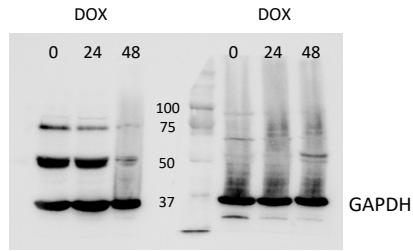

2D

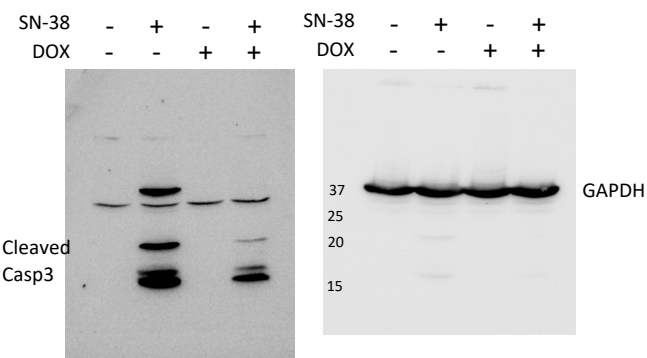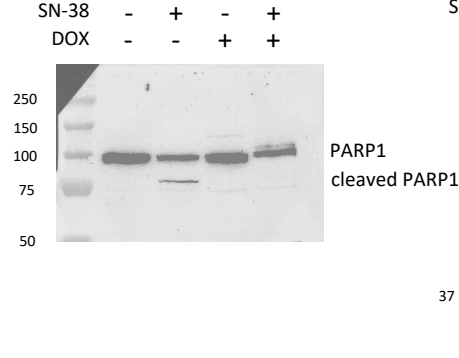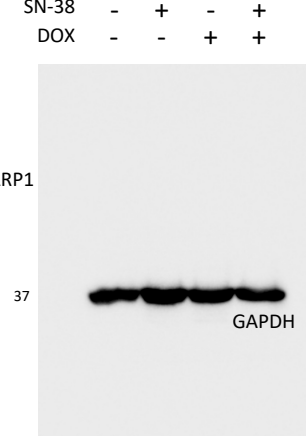

2I

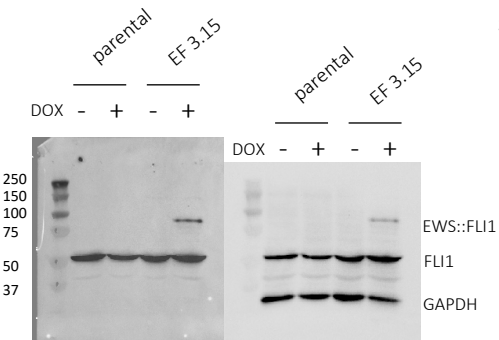

S2F

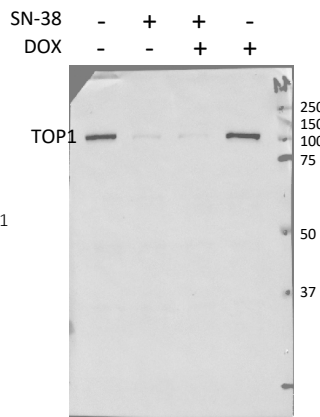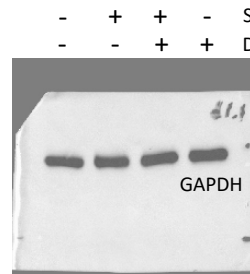

S2G

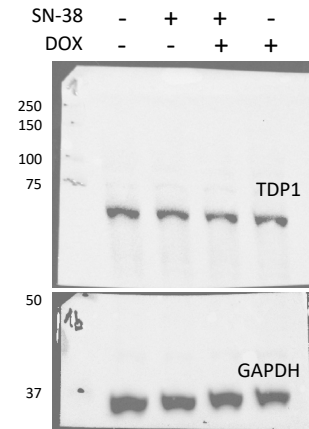

S2G

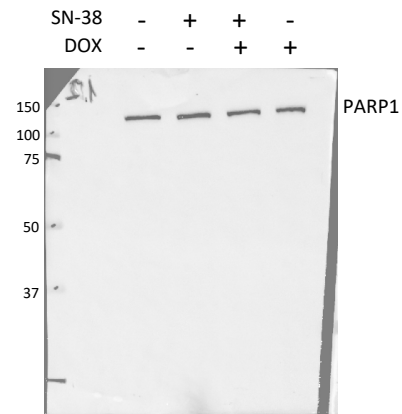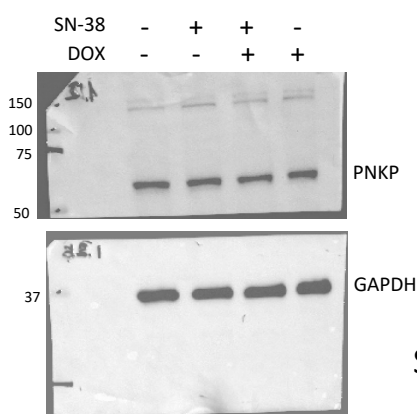

S3F

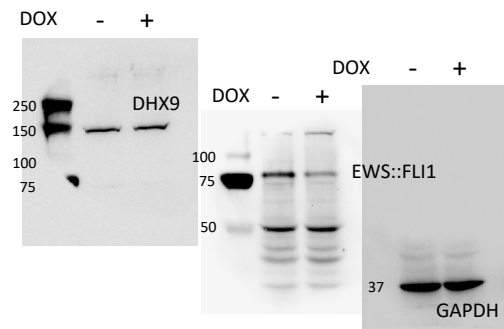

S3G

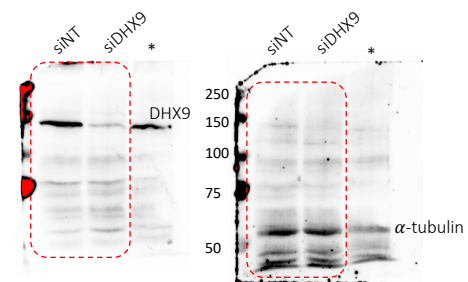

4B

R1

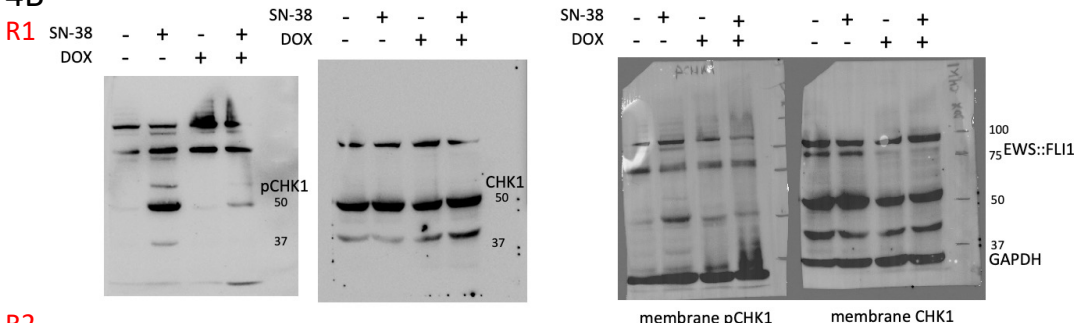

R2

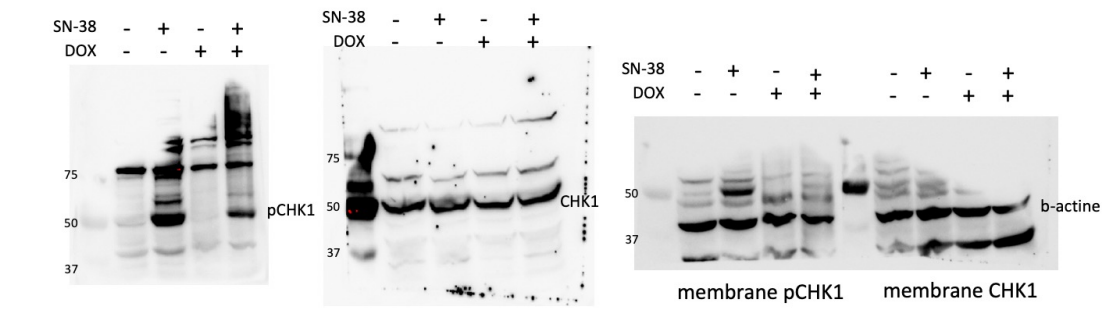

4E

R1

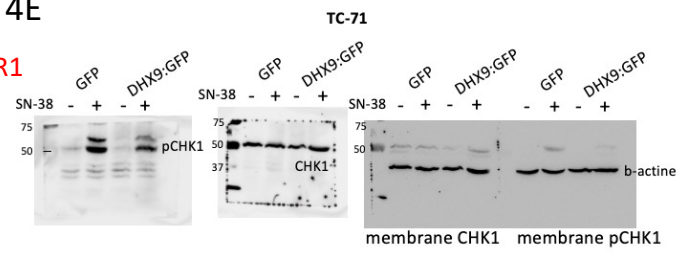

R2

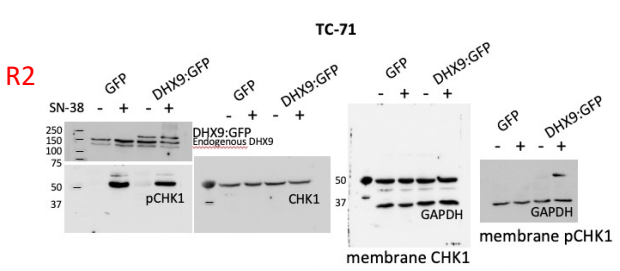

R1

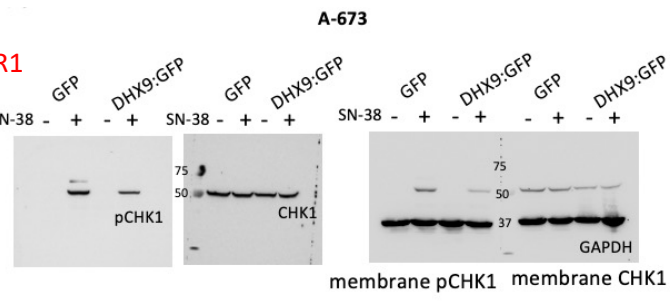

R2

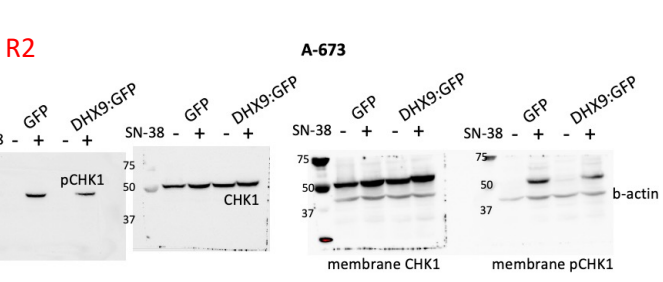

4F

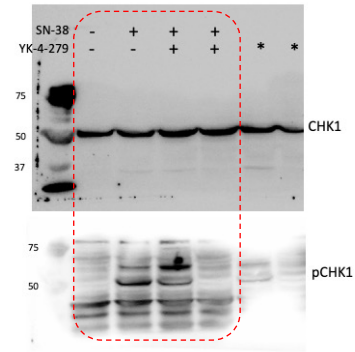

4G

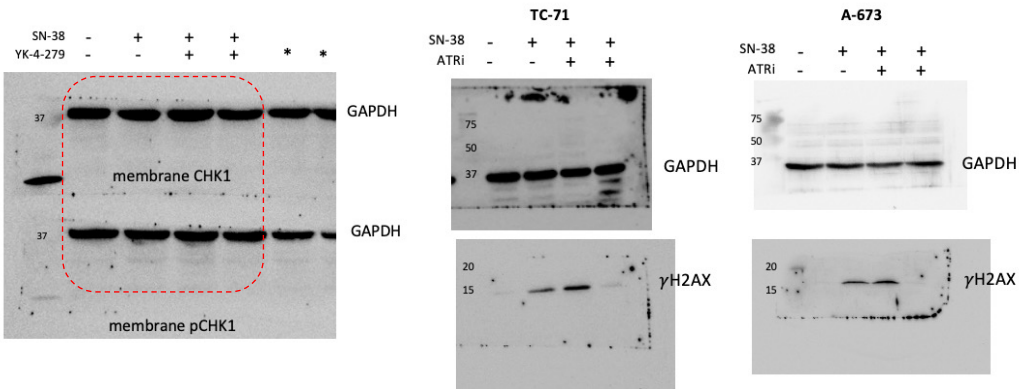

4G

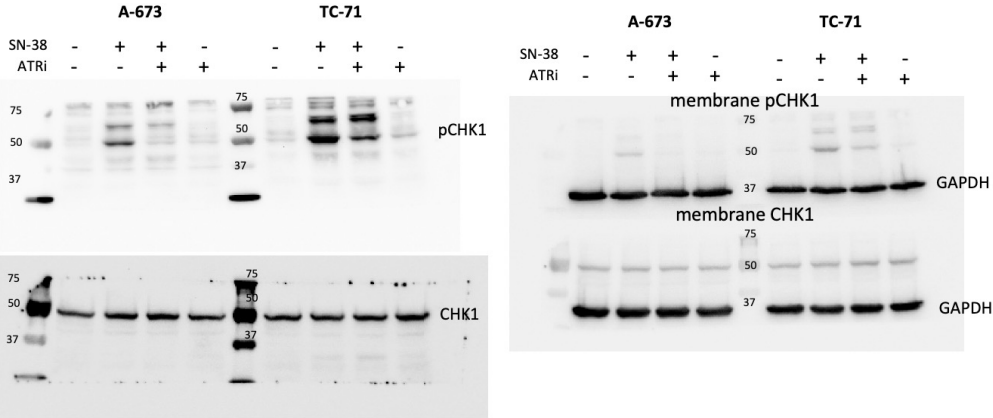

S4A

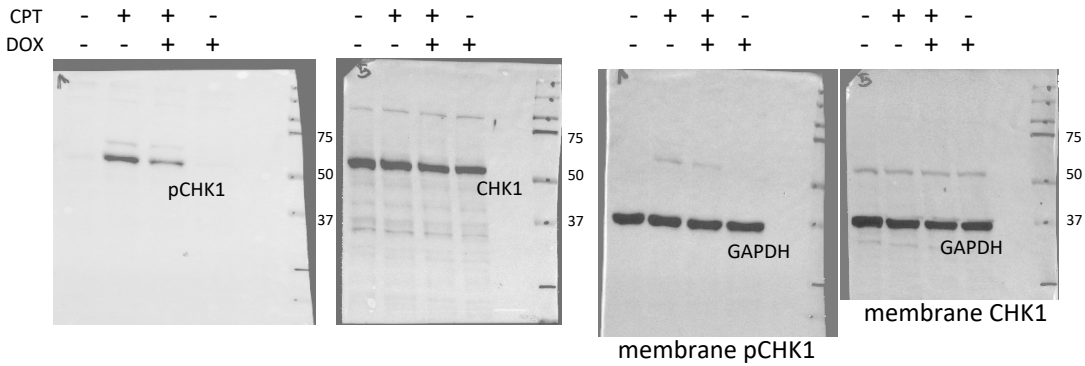

S4C

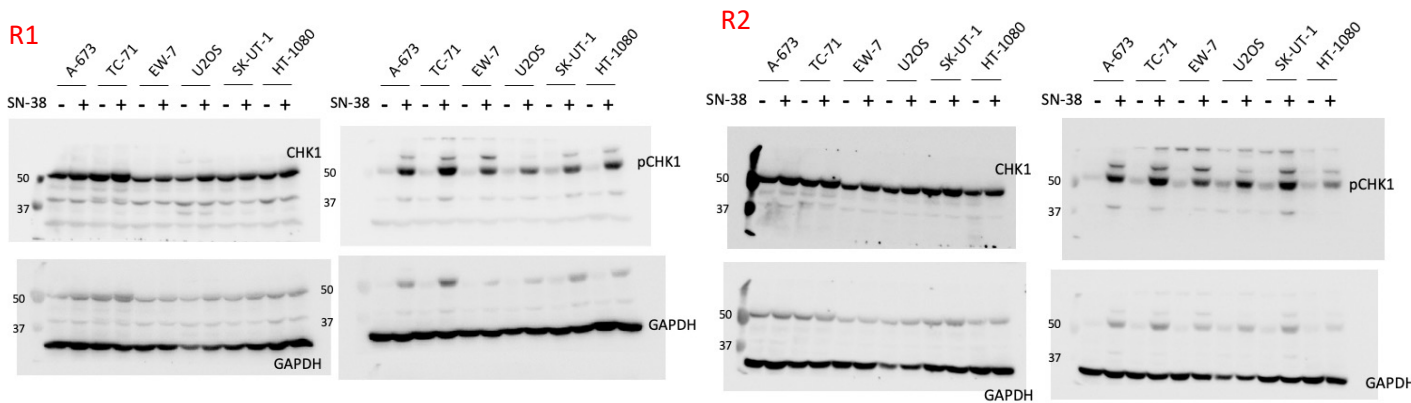

5A

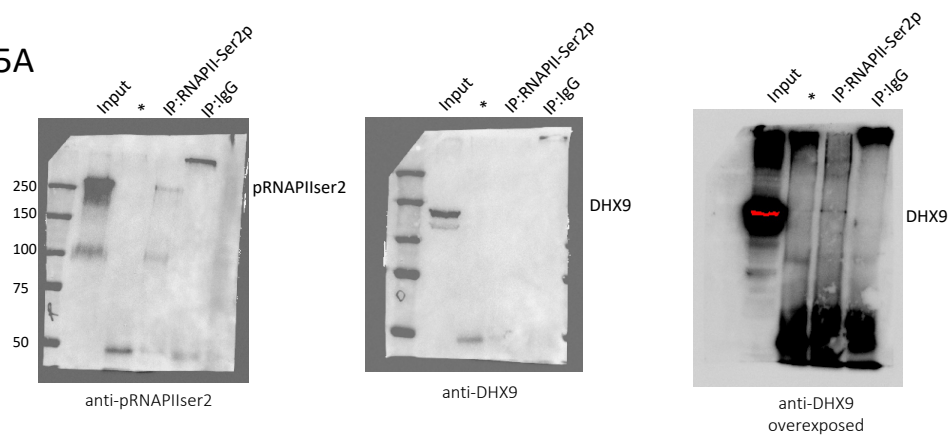

5B

R1

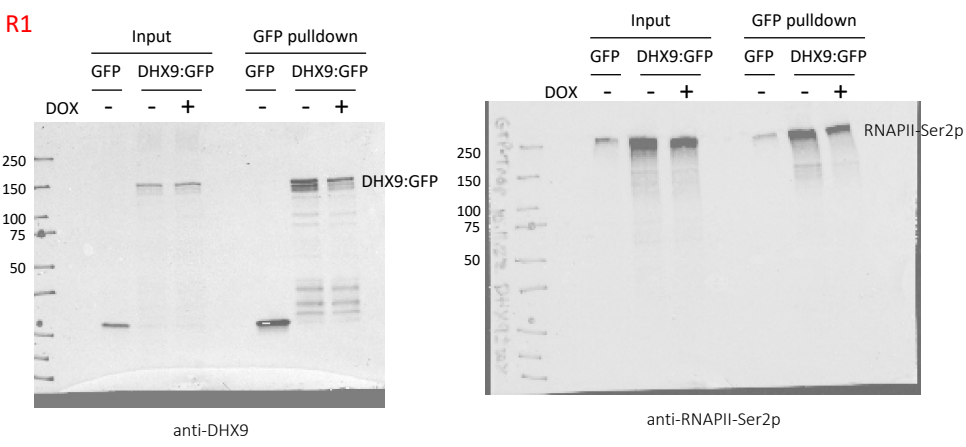

R2

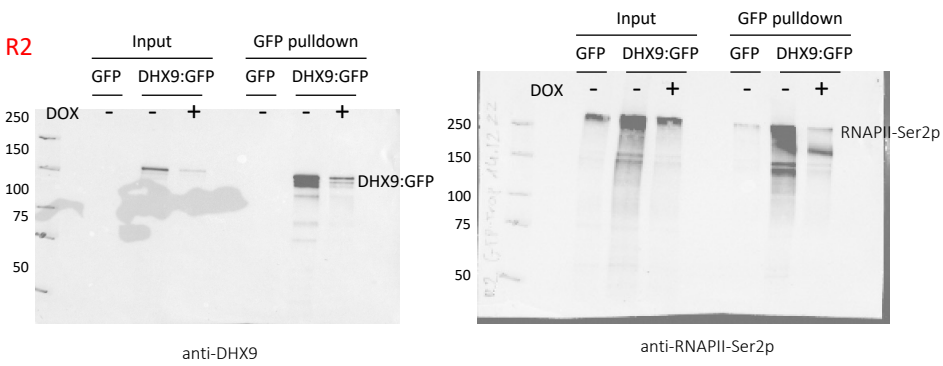

R3

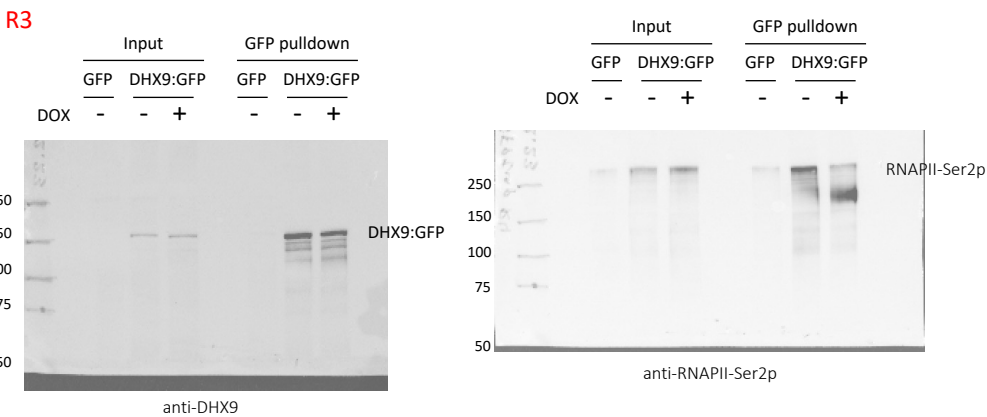

5E

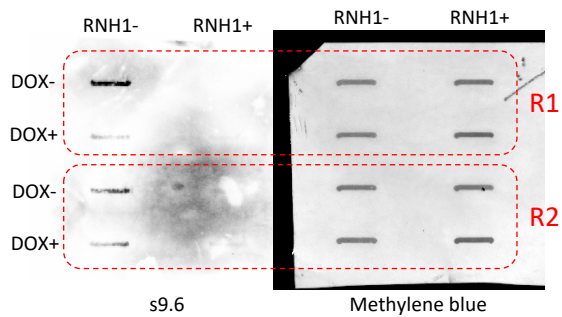

R3

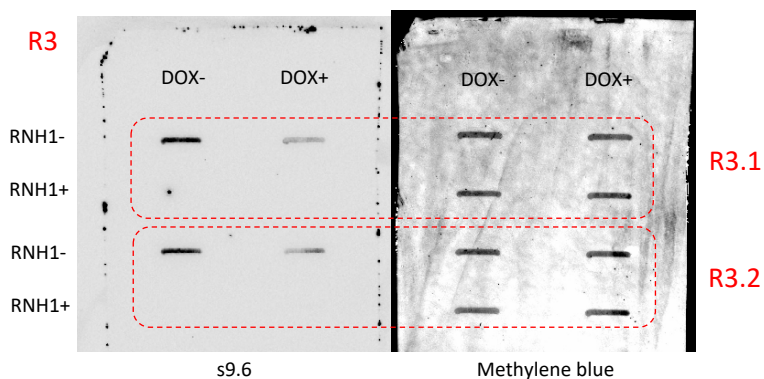

5G

R1

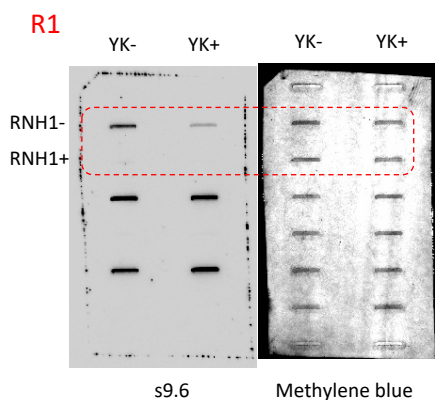

R2

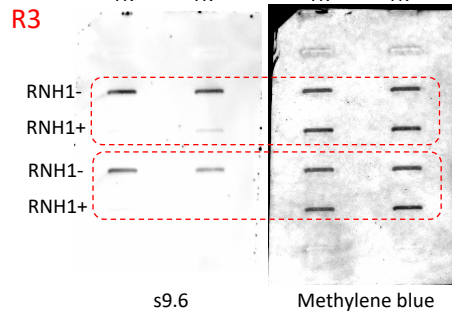

R4

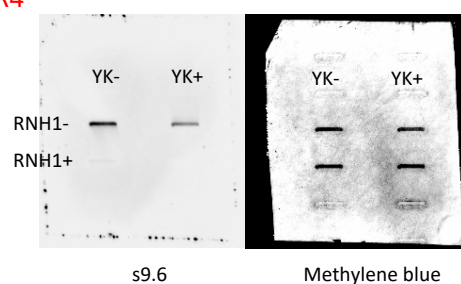

5I

R1

R2

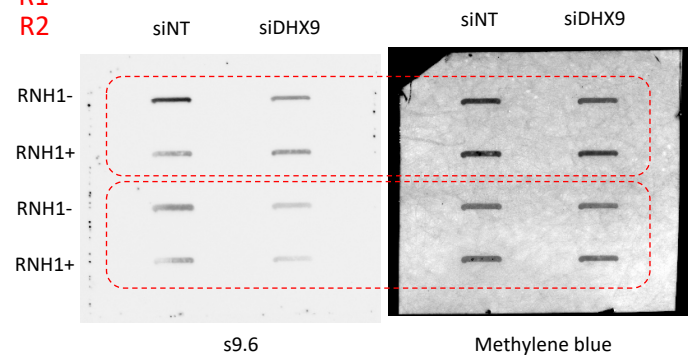

R3

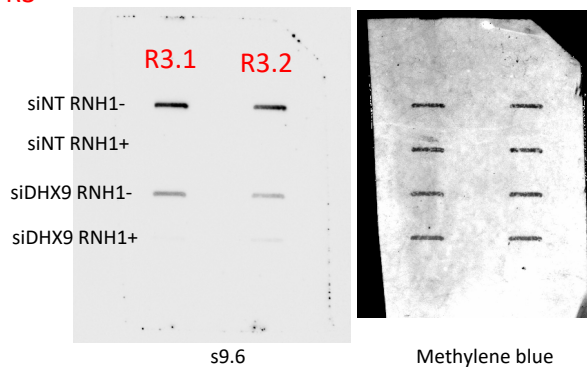

S5F

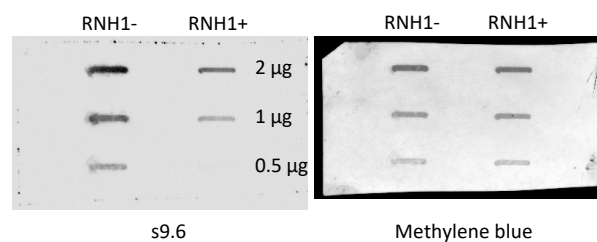

Supplement: Supplementary file 1 — uncropped blots [file 41388_2025_3496_MOESM1_ESM.pdf]
