# Supplementary material for: EWS::FLI1-DHX9 interaction promotes Ewing sarcoma sensitivity to DNA topoisomerase 1 poisons by altering R-loop metabolism
Source: Oncogene. 2025 Jul 28;44(38):3537–52. doi: 10.1038/s41388-025-03496-9 (PMC12436182; doi:10.1038/s41388-025-03496-9)
Supplement: Supplementary file 2 — Supplementary Figures [file 41388_2025_3496_MOESM2_ESM.pdf]

SUPPLEMENTARY FIGURES.

A

Drug activity
(EwS (*EWSR1::FLI1*+) vs. non-EwS cells)
Genomics of Drug Sensitivity in Cancer (GDSC2)

Drug	Target	Drug effect	P-value
Irinotecan	TOP1	-1.57	$4.48e^{-8}$
Topotecan	TOP1	-1.2	$5.03e^{-5}$
Camptothecin	TOP1	-1.11	$6.31e^{-5}$
SN-38	TOP1	-0.93	0.006
LMP744	TOP1	-1.11	0.0002

B

Model	Disease	# of tumors	CR	PR	SD	PD
IEC-073	EwS	6	100% (6/6)	0	0	0
HSJD-ES-002	EwS	8	87,5% (7/8)	12,5% (1/8)	0	0
HSJD-ES-006	EwS	6	100% (6/6)	0	0	0
IEC-036	OS	4	0	25% (1/4)	75% (3/4)	0
IEC-056	UPS	4	0	0	100% (4/4)	0

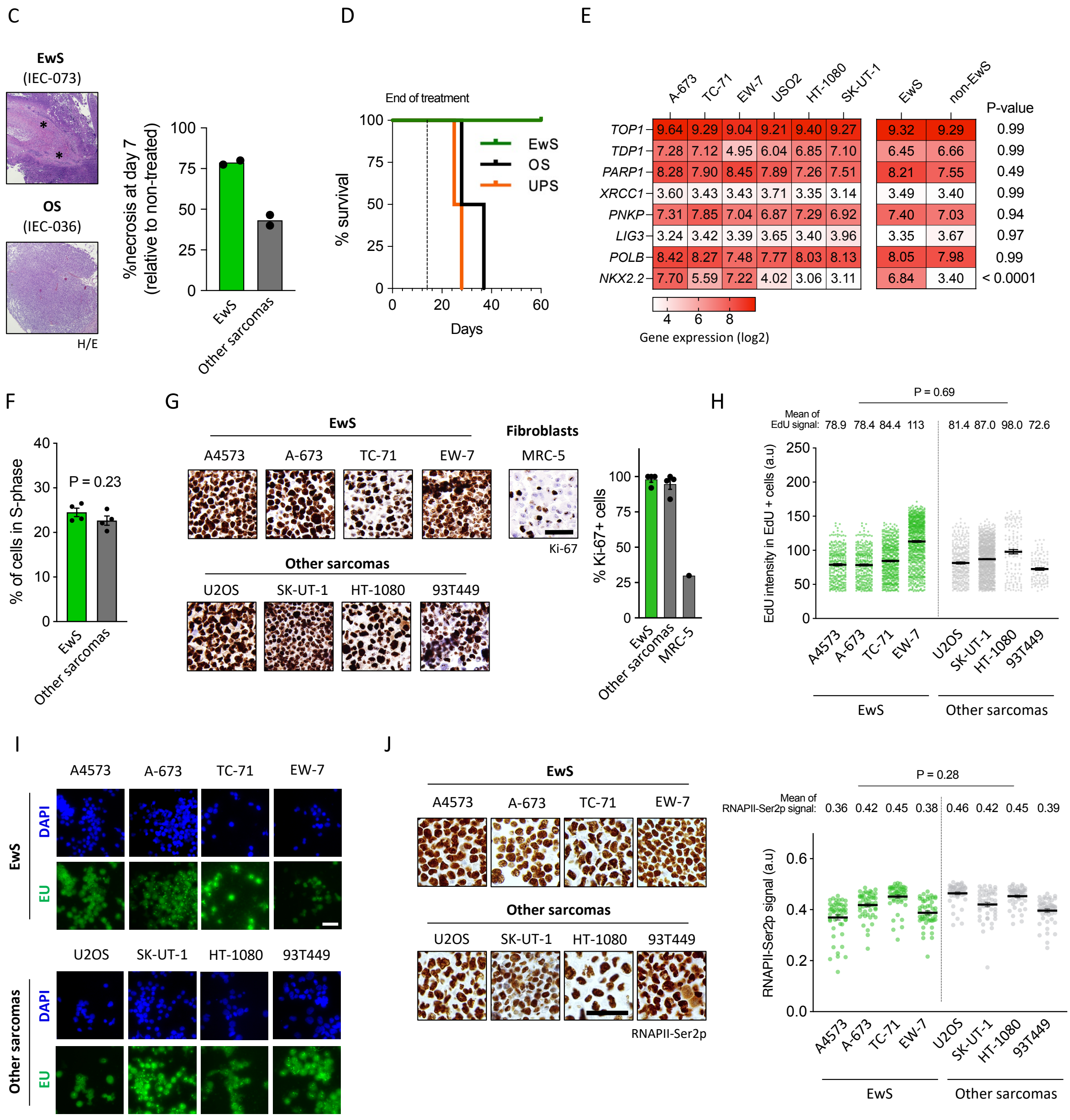

Supplementary Figure 1. **(A)** Effect of TOP1 poisons and inhibitors between EwS (carrying *EWSR1::FLI1* fusion oncogene) and non-EwS cell lines. Data were obtained from Genomics of Drug Sensitivity in Cancer database. **(B)** Evaluation of the response to irinotecan treatment of EwS and non-EwS PDXs 21 days after the beginning (CR, complete response; PR, partial response; SD, stable disease; PD, progressive disease). **(C)** Evaluation of tumor necrosis at day 7 of treatment. *Left*, representative hematoxylin/eosin images. Asterisks indicate necrotic areas. *Right*, data represent the mean of the percentage of tumoral necrosis. **(D)** Kaplan-Meier curves comparing survival of PDXs models treated with irinotecan. **(E)** Expression levels of *TOP1, TDP1,* and single-strand break repair factors between EwS and non-EwS cells using public expression data (E-MTAB-3610). *NKX2.2*, an EWS::FLI1 transcriptional target was included as a positive control. **(F)** Analysis of S-phase population between EwS and non-EwS cell lines by FACS (propidium iodide staining). Data represent the mean (±SEM) of the percentage of cells in S-phase. **(G)** Evaluation of proliferation of EwS and non-EwS cell lines by Ki-67 ICC in paraffin-embedded pellets. *Left,* representative images. Scale bar, 50 µm. *Right,* data represent the mean (±SEM) of the percentage of Ki-67-positive cells. **(H)** Analysis of replication efficiency between EwS and non-EwS cells by EdU incorporation. Data represent the mean (±SEM) of nuclear EdU intensity in EdU-positive cells (a total of approximately 100 cells were analyzed). **(I)** Evaluation of EU incorporation between EwS and non-EwS cells. Representative images of data shown in Fig. 1G. DAPI counterstain. Scale bar, 50 µm. **(J)** Evaluation of transcriptional activity of EwS and non-EwS cell lines by RNAPII-Ser2p ICC in paraffin-embedded pellets. *Left,* representative images. Scale bar, 50 µm. *Right,* data represent the mean (±SEM) of RNAPII-Ser2p signal. P-value was determined by t-test.

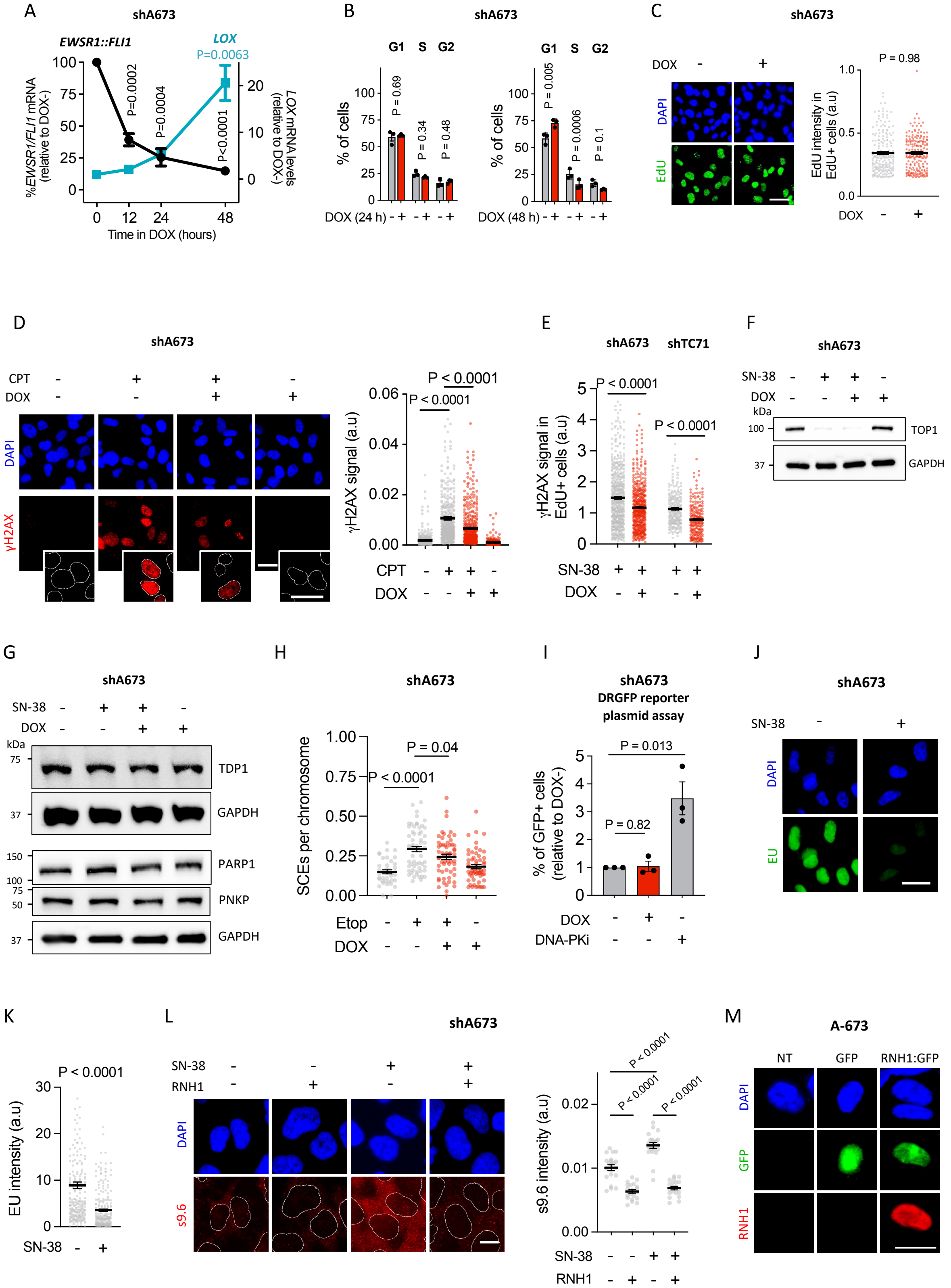

A
shA673
EWSR1::FLI1
LOX
P=0.0063
P=0.0002
P=0.0004
P<0.0001
%EWSR1/FLI1 mRNA (relative to DOX-)
LOX mRNA levels (relative to DOX-)
Time in DOX (hours)
B
shA673
G1 S G2
% of cells
P = 0.69
P = 0.34
P = 0.48
DOX (24 h)
P = 0.005
P = 0.0006
P = 0.1
DOX (48 h)
C
shA673
DOX
DAPI
EdU
P = 0.98
EdU intensity in EdU+ cells (a.u)
D
shA673
CPT
DOX
DAPI
γH2AX
P < 0.0001
γH2AX signal (a.u)
E
shA673
shTC71
γH2AX signal in EdU+ cells (a.u)
SN-38
F
shA673
kDa
TOP1
GAPDH
G
shA673
TDP1
GAPDH
PARP1
PNKP
H
shA673
P = 0.04
SCEs per chromosome
Etop
I
shA673
DRGFP reporter plasmid assay
P = 0.013
P = 0.82
% of GFP+ cells (relative to DOX-)
DNA-PKi
J
shA673
EU
K
P < 0.0001
EU intensity (a.u)
L
shA673
RNH1
s9.6
s9.6 intensity (a.u)
M
A-673
NT
GFP
RNH1:GFP
RNH1

Supplementary Figure 2. **(A)** Evaluation by qPCR of *EWSR1::FLI1* and *LOX* mRNA levels in shA673 cells after incubation with DOX for the indicated hours. Data represent the mean (±SEM) of gene expression (normalized to GAPDH, relative to DOX-); n=3 independent experiments. **(B)** Analysis of cell cycle of shA673 by PI FACS. Data represent the mean (±SEM) of cell cycle populations after incubation with DOX for indicated hours; n=3 independent experiments. **(C)** Determination of replication activity in shA673 cells after 24 h of DOX incubation by EdU incorporation. *Left*, representative images. DAPI counterstain. Scale bar, 50 μm. *Right*, data represent the mean (±SEM) of nuclear EdU intensity in EdU+ cells, n=2 independent experiments (100 cells were analyzed per replicate). **(D)** CPT-induced γH2AX upon *EWSR1::FLI1* knockdown by IF. Cells were pre-incubated with DOX and treated with 5 μM CPT (30 min). *Left*, representative images. DAPI counterstain. Scale bar, 20 μm. *Right*, data represent the mean (±SEM) of nuclear γH2AX intensity, n=3 independent experiments (100 cells were analyzed per replicate). **(E)** Evaluation of DSBs by γH2AX IF in S-phase population, determined by EdU incorporation. shA673 and shTC71 cells were incubated with DOX for 24 h. Then, cells were incubated with 10 μM of EdU (20 min), washed and treated with 5 μM SN-38 for 30 min. Data represent the mean (±SEM) of nuclear γH2AX intensity in EdU+ cells, n≥2 independent experiments (100 cells were analyzed per replicate). **(F)** Analysis of TOP1 and **(G)** TDP1, PARP1 and, PNKP protein levels in shA673 cells after incubation with DOX for 24 h and treatment with 5 μM SN-38 (30 min) by WB. Loading control: GAPDH. Molecular weight in kDa. **(H)** Study of HR efficiency by SCEs assay in shA673 model upon *EWSR1::FLI1* knockdown. Cells were incubated with DOX for 24 h and treated with 2.5 μM etoposide (30 min). Data represent the mean (±SEM) of SCEs; n=3 independent experiments (approximately 20 metaphases were analyzed per replicate). **(I)** Analysis of HR efficiency by DRGFP reporter plasmid assay in shA673 cells upon *EWSR1::FLI1* knockdown. Where is indicated, cells were treated with DOX or 10 μM of DNA-PK inhibitor NU-7441 for 24 h. Data represent the mean (±SEM) of the percentage of GFP-positive cells (normalized to mCherry-positive cells, used as a control of transfection, and relative to DOX-); n=3 independent experiments. **(J)** Evaluation of transcription by EU incorporation. shA673 cells were treated with 5 μM SN-38 (30 min). Representative images. DAPI counterstain. Scale bar, 20 μm. **(K)** Quantification of (J). Data represent the mean (±SEM) of EU signal, n=3 independent experiments (approximately 75 cells were analyzed per replicate). **(L)** Evaluation of R-loops levels in shA673 cells after treatment with 5 μM SN-38 (30 min) and *in vitro* incubation with RNH1. *Left,* representative images. DAPI counterstain. Scale bar, 20 μm. *Right,* data represent the mean (±SEM) of nuclear s9.6 intensity (20 cells were analyzed). **(M)** Evaluation of RNH1 levels in A-673 cells after 24 h of transfection with RNH1:GFP or control plasmids.

Representative images. DAPI counterstain. Scale bar, 20 μm. P-value was determined by t-test.

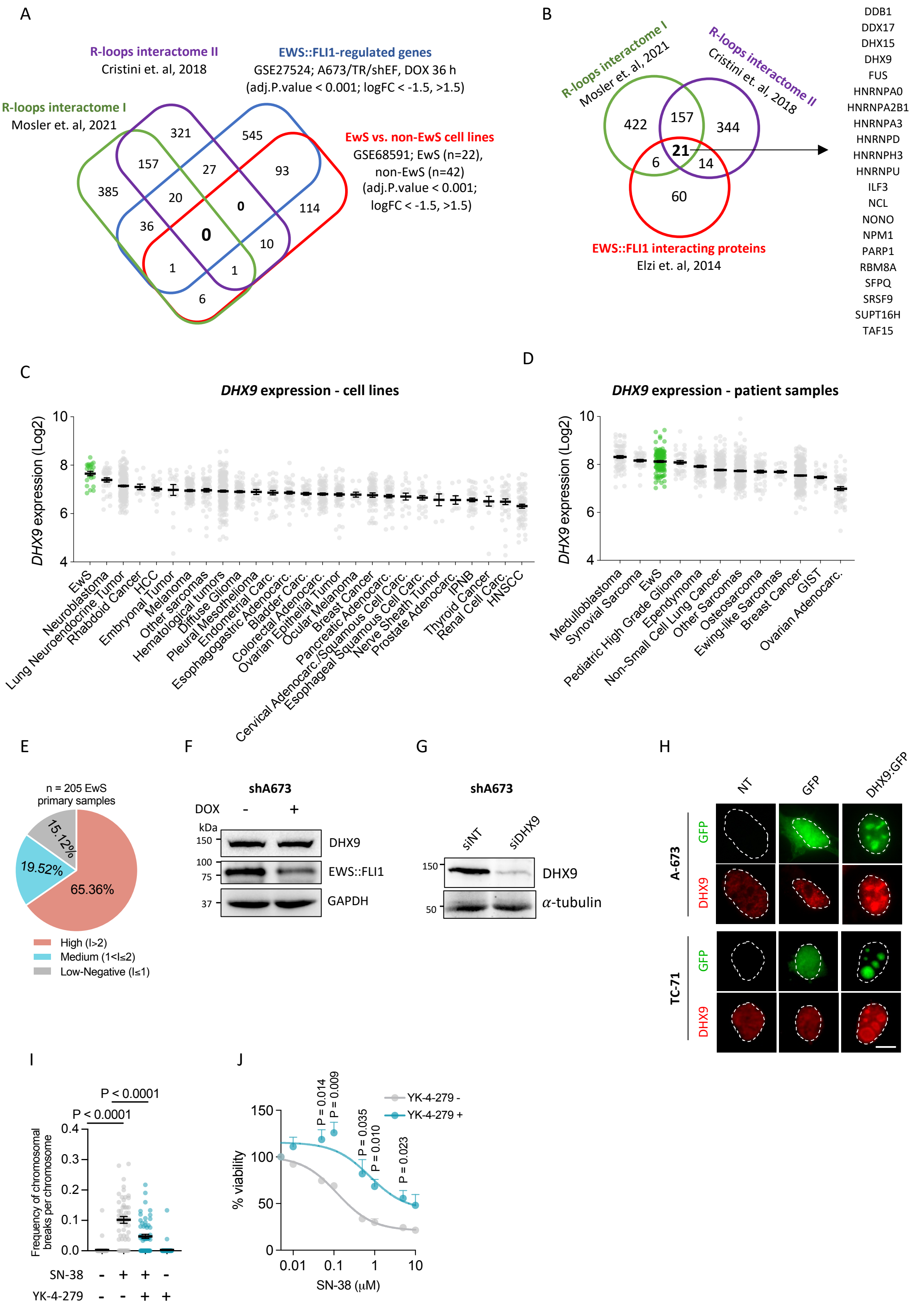
A
R-loops interactome II
Cristini et. al, 2018
EWS::FLI1-regulated genes
GSE27524; A673/TR/shEF, DOX 36 h
(adj.P.value < 0.001; logFC < -1.5, >1.5)
R-loops interactome I
Mosler et. al, 2021
EwS vs. non-EwS cell lines
GSE68591; EwS (n=22),
non-EwS (n=42)
(adj.P.value < 0.001;
logFC < -1.5, >1.5)
321
545
157
27
93
385
20
0
114
36
0
10
1
1
6
B
R-loops interactome I
Mosler et. al, 2021
R-loops interactome II
Cristini et. al, 2018
422
157
344
21
6
14
60
EWS::FLI1 interacting proteins
Elzi et. al, 2014
DDB1
DDX17
DHX15
DHX9
FUS
HNRNPA0
HNRNPA2B1
HNRNPA3
HNRNPD
HNRNPH3
HNRNPU
ILF3
NCL
NONO
NPM1
PARP1
RBM8A
SFPQ
SRSF9
SUPT16H
TAF15
C
DHX9 expression - cell lines
DHX9 expression (Log2)
10
8
6
4
EwS
Neuroblastoma
Lung Neuroendocrine Tumor
Rhabdoid Cancer
HCC
Embryonal Tumor
Melanoma
Other sarcomas
Hematological tumors
Diffuse Glioma
Pleural Mesothelioma
Endometrial Carc.
Esophagogastric Adenocarc.
Bladder Carc.
Colorectal Adenocarc.
Ovarian Epithelial Tumor
Ocular Melanoma
Breast Cancer
Pancreatic Adenocarc.
Cervical Adenocarc./Squamous Cell Carc.
Esophageal Squamous Cell Carc.
Nerve Sheath Tumor
Prostate Adenocarc.
IPNB
Thyroid Cancer
Renal Cell Carc.
HNSCC
D
DHX9 expression - patient samples
DHX9 expression (Log2)
10
8
6
4
Medulloblastoma
Synovial Sarcoma
EwS
Pediatric High Grade Glioma
Ependymoma
Non-Small Cell Lung Cancer
Other Sarcomas
Osteosarcoma
Ewing-like Sarcomas
Breast Cancer
GIST
Ovarian Adenocarc.
E
n = 205 EwS
primary samples
15.12%
19.52%
65.36%
High (I>2)
Medium (1<I≤2)
Low-Negative (I≤1)
F
shA673
DOX
-
+
kDa
150
100
75
37
DHX9
EWS::FLI1
GAPDH
G
shA673
siNT
siDHX9
150
50
DHX9
α-tubulin
H
NT
GFP
DHX9:GFP
A-673
TC-71
GFP
DHX9
I
P < 0.0001
P < 0.0001
Frequency of chromosomal breaks per chromosome
0.4
0.3
0.2
0.1
0.0
SN-38
YK-4-279
J
P = 0.014
P = 0.009
P = 0.035
P = 0.010
P = 0.023
YK-4-279 -
YK-4-279 +
150
100
50
0
% viability
0.01
0.1
1
10
SN-38 (μM)

Supplementary Figure 3. **(A)** Venn diagram between EWS::FLI1 transcriptionally regulated genes (GSE27524), differentially expressed genes between EwS and non-EwS sarcoma cell lines (GSE68591) and R-loop interactomes. **(B)** Similar to (A) between R-loop and EWS::FLI1 interactomes. **(C)** *DHX9* expression levels in tumoral cell lines (HCC: hepatocellular carcinoma; IPNB: intraductal papillary neoplasia of the bile duct; HNSCC: head and neck squamous cell carcinoma). **(D)** *DHX9* expression levels in tumor samples (GIST: gastrointestinal stromal tumor). **(E)** Evaluation of DHX9 protein levels in a cohort of 205 EwS primary tumors by IHC. Samples were grouped according to DHX9 signal (High: I>2; Medium: I>1 and ≤2; Low: I≤1; Negative: I=0). **(F)** Evaluation of the effect of *EWSR1::FLI1* downregulation on DHX9 protein levels by WB. shA673 cells were incubated with DOX for 24 h. Loading control: GAPDH. Molecular weight in kDa. **(G)** Determination of DHX9 protein levels after 48 h of transfection with indicated siRNAs. Loading control: α-tubulin. Molecular weight in kDa. **(H)** Evaluation of DHX9 protein levels in A-673 and TC-71 cells after transfection with DHX9:GFP or control plasmids by IF. Representative images. DAPI counterstain. Scale bar, 10 µm. **(I)** Effect of YK-4-279 on SN-38-induced chromosomal breaks. A-673 cells were pre-incubated with 75 µM YK-4-279 (1.5 h) and treated with 2.5 µM SN-38 (30 min). Data represent the mean (±SEM) of chromosomal breaks per chromosome, n=3 independent experiments (20 metaphases were analyzed per replicate). **(J)** Evaluation of the effect of YK-4-279 in cell survival upon SN-38 exposure. A-673 cells were pre-incubated with 75 µM YK-4-279 (1.5 h) and treated with indicated concentrations of SN-38 (3 h). After treatment, cells were cultured in drug-DOX-free medium for 48 h previous MTT assay. Data represent the mean (+SEM) of the percentage of survival, n=3 independent experiments. P-value was determined by t-test.

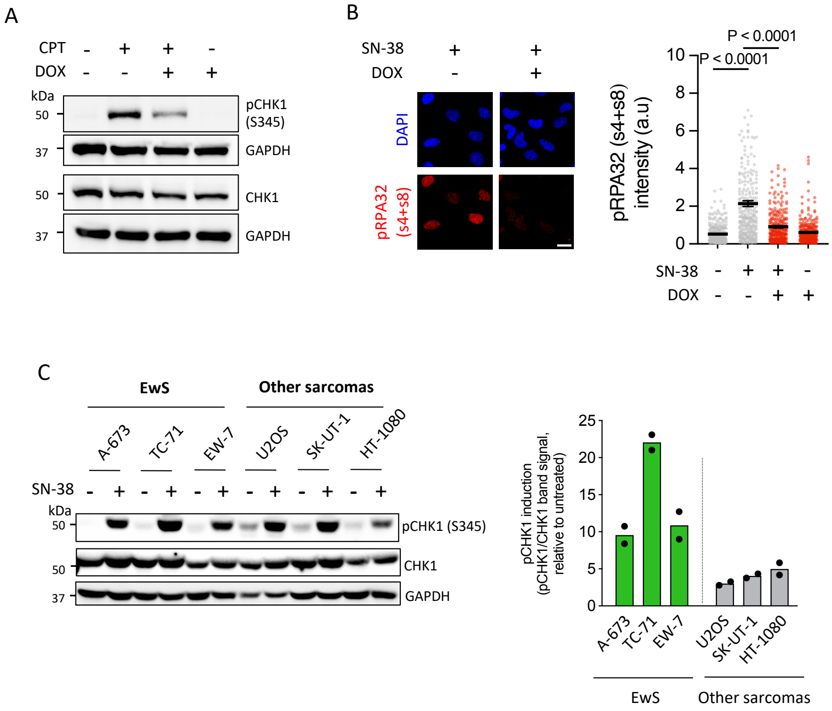

D

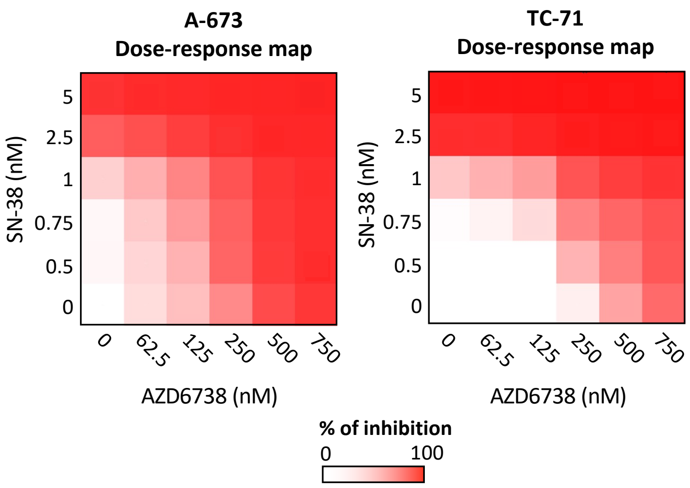

E

	SN-38 IC50 (nM)		
	**AZD6738 -**	**AZD6738 +**	**SN-38 IC50 (AZD6728-/+)**
**A-673**	2.22	1.36	**1.63x**
**TC-71**	1.59	0.89	**1.78x**

Supplementary Figure 4. (A) Effect of *EWSR1::FLI1* knockdown in CPT-induced replication stress by pCHK1 (Ser345) WB. shA673 cells were pre-incubated with DOX for 24 h and treated with 5 µM CPT (30 min). Loading control: GAPDH. Molecular weight in kDa. **(B)** Analysis of the effect of *EWSR1::FLI1* knockdown on SN-38-induced pRPA32 (s4+s8) by IF. *Left*, representative images. DAPI counterstain. Scale bar, 20 µm. *Right,* data represent the mean (±SEM) of pRPA32 (s4+s8) intensity, n=3 independent experiments (100 cells were analyzed per replicate). **(C)** Evaluation of SN-38-induced pCHK1 levels between EwS and non-EwS cell lines. *Left*, representative immunoblots. Details as in (A). *Right,* data represent the mean of pCHK1 induction (pCHK1/CHK1 band signal, relative to untreated), n=2 independent experiments. **(D)** Dose-response matrix of the combination between AZD6738 and SN-38 in A-673 and TC-71 cell lines. **(E)** IC50 data from MTT assay. Cells were incubated with AZD6738 (250 nM, 24 h) previous to the treatment with SN-38 (72 h). P-value was determined by t-test.

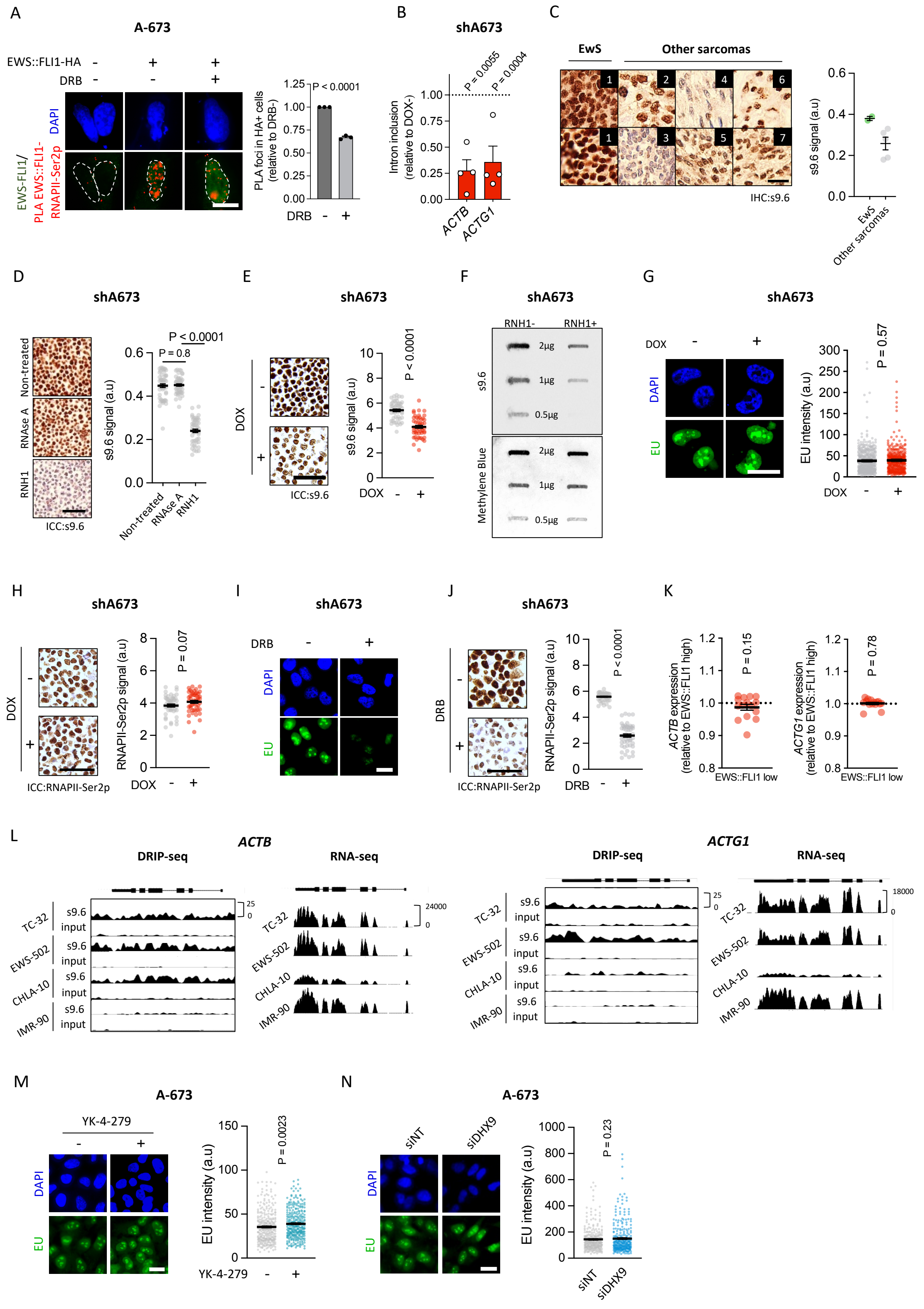
A
A-673
EWS::FLI1-HA
DRB
DAPI
EWS-FLI1/
PLA EWS::FLI1-
RNAPII-Ser2p
PLA foci in HA+ cells (relative to DRB-)
P < 0.0001
B
shA673
Intron inclusion (relative to DOX-)
P = 0.0055
P = 0.0004
ACTB
ACTG1
C
EwS
Other sarcomas
IHC:s9.6
s9.6 signal (a.u)
D
shA673
Non-treated
RNAse A
RNH1
ICC:s9.6
P < 0.0001
P = 0.8
E
shA673
DOX
P < 0.0001
s9.6 signal (a.u)
F
shA673
RNH1-
RNH1+
s9.6
Methylene Blue
2µg
1µg
0.5µg
G
shA673
DOX
DAPI
EU
EU intensity (a.u)
P = 0.57
H
shA673
ICC:RNAPII-Ser2p
RNAPII-Ser2p signal (a.u)
P = 0.07
I
shA673
DRB
J
shA673
P < 0.0001
K
ACTB expression (relative to EWS::FLI1 high)
ACTG1 expression (relative to EWS::FLI1 high)
EWS::FLI1 low
P = 0.15
P = 0.78
L
ACTB
ACTG1
DRIP-seq
RNA-seq
TC-32
EWS-502
CHLA-10
IMR-90
s9.6
input
M
A-673
YK-4-279
P = 0.0023
N
A-673
siNT
siDHX9
P = 0.23

Supplementary Figure 5. (A) Analysis of EWS::FLI1-RNAPII-Ser2p interactions by PLA using anti-HA and anti-RNAPII-Ser2p antibodies. shA673 cells overexpressing EWS::FLI1-HA were treated with 100 µM DRB (2 h). *Left*, representative images (red, PLA foci; green, EWS::FLI1-HA overexpression; blue, DAPI counterstain). Scale bar, 10 µm. *Right*, data represent the mean (+SEM) of PLA foci number per cell (relative to DRB-), n=3 independent experiments. **(B)** Analysis of the effect of *EWSR1::FLI1* downregulation in mRNA splicing by qPCR. shA673 cells were incubated with DOX for 24 h. Data represent the mean (+SEM) of the rate of intron inclusion in DOX-treated cells (relative to untreated), n=4 independent experiments. **(C)** Evaluation of R-loops levels in a sarcoma tissue microarray by s9.6 IHC. *Left*, representative images (1. EwS; 2. Osteosarcoma; 3. Synovial sarcoma; 4. GIST; 5. Embryonal Rhabdomyosarcoma; 6. Leiomyosarcoma; 7. Undifferentiated Liposarcoma). Scale bar, 20 µm. *Right,* quantification of R-loops levels. **(D)** Similar to (C) in shA673 paraffin-embedded cellular pellets after *in vitro* treatment with RNAse A or RNH1. *Left*, representative images. Scale bar, 50 µm. *Right*, data represent the mean (±SEM) of s9.6 signal. **(E)** Similar to (D) after incubation with DOX for 24 h. **(F)** s9.6 slot blot. shA673 extracts were treated *in vitro* with RNH1 and loaded at different concentrations. Membranes were incubated with s9.6 antibody and stained with methylene blue. **(G)** Analysis of the effect of *EWSR1::FLI1* knockdown in global transcription by EU incorporation. shA673 cells were incubated with DOX for 24 h. *Left*, representative images. DAPI counterstain. Scale bar, 20 µm. Right, data represent the mean (±SEM) of nuclear EU intensity, n=3 independent experiments (150 cells were analyzed per replicate). **(H)** Similar to (G) by RNAPII-Ser2p ICC. *Left*, representative images. Scale bar, 50 µm**.** *Right*, data represent the mean (±SEM) of RNAPII-Ser2p signal. **(I)** Similar to (G) after treatment with 100 µM DRB for 1 h. **(J)** Similar to (I) by RNAPII-Ser2p ICC. **(K)** Analysis of the expression levels of *ACTB* and *ACTG1* genes in 15 EwS cell lines upon *EWSR1::FLI1* knockdown using a public dataset (GSE176190). Data represent the mean (±SEM) of mRNA levels in EWS::FLI1 low condition (relative to EWS::FLI1 high). **(L)** Evaluation of DRIP (GSE68845) and RNA-seq (GSE68836) signal in *ACTB* and *ACTG1* genes between EwS (TC-32, EWS-502 and CHLA-10) and non-EwS IMR-90 cell lines. **(M)** Analysis of the effect of YK-4-279 treatment in global transcription by EU incorporation. A-673 cells were treated with 75 µM YK-4-279 for 1.5 h. *Left*, representative images. DAPI counterstain. Scale bar, 20 µm. Right, data represent the mean (±SEM) of nuclear EU intensity, n=2 independent experiments (150 cells were analyzed per replicate). **(N)** Similar to (M) after 48 h of transfection with indicated siRNAs. P-value was determined by t-test.
